# Transformations of Head Structures During the Larval Development of the Black Soldier Fly *Hermetia illucens* (Stratiomyidae, Diptera)

**DOI:** 10.1002/jmor.70048

**Published:** 2025-04-11

**Authors:** Benjamin Fabian, Katharina Schneeberg, Stephan Löwe, René Bauernfeind, Rolf Georg Beutel

**Affiliations:** ^1^ Research Group Olfactory Coding, Max‐Planck‐Institut für Chemische Ökologie Jena Germany; ^2^ Pfalzmuseum für Naturkunde Bad Dürkheim Germany; ^3^ Lehrstuhl für Zoologie I, Zell‐ und Entwicklungsbiologie Julius‐Maximilians‐Universität Würzburg Würzburg Germany; ^4^ August‐Bebel‐Straße 1 Kahla Germany; ^5^ Institut für Zoologie und Evolutionsforschung Friedrich‐Schiller‐Universität Jena Jena Germany

**Keywords:** head anatomy, instars, maggots, postembryonic development, soldier flies

## Abstract

Larvae of the black soldier fly, *Hermetia illucens*, are currently intensively studied, owing to their potential importance in various fields such as waste bioconversion, forensic entomology, and food supply for humans and life stock. Despite the increased attention, a detailed anatomical documentation of the larvae using modern methods is lacking, and even statements on the number of larval stages are contradictory. Misinterpretations of the ontogeny of this species have led to frequent erroneous identifications of the last larval instar as pupa. Consequently, many studies with a focus on larval morphology have neglected the last larval stage. In this contribution, we describe and document morphological changes of the larval head throughout the postembryonic development, with emphasis on the transition between the last two instars. This is characterized by a crucial behavioral shift from a feeding stage to a stage of increased vagility. We show that different cephalic structures undergo major changes, especially the mandibulo‐maxillary complex and the digestive tract, and associated muscles. Our measurements of the body length and the length of the head capsule tentatively confirm that the larval development of *H. illucens* passes through seven instars.

## Graphical abstract

The black soldier fly (*Hermetia illucens*) has seven larval instars. Drain channels of the digestive system, metacephalic rods, and the cephalic musculature are described for the 1st time. Profound changes take place between the stages 6 and 7.

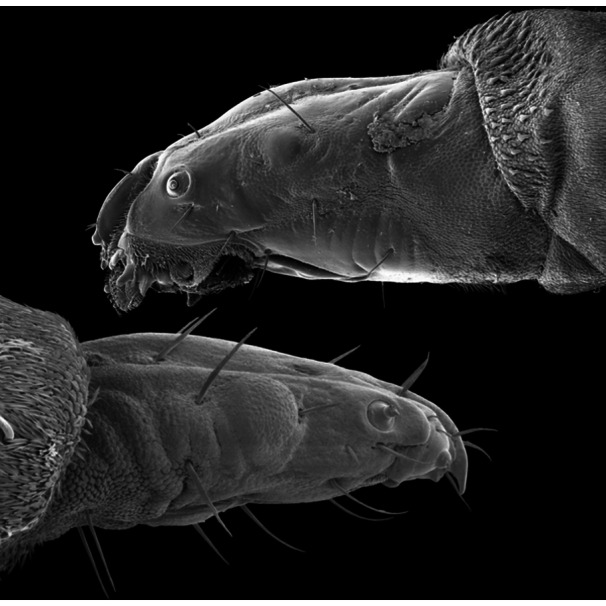

## 1 Introduction

The black soldier fly *Hermetia illucens*) is a species that is currently studied intensively, especially the saprophagous larvae. The immature stages play a role in forensic entomology (Lord et al. 1994), in pest control (Furman et al. 1959), in composting and bioconversion (Lardé 1990; Lalander et al. 2013; Banks 2014; Gold et al. 2018; Mertenat et al. 2019; Surendra et al. 2020), and also as food for humans and various animals, including life stock and pets (Newton et al. 1977; Bondari and Sheppard 1987; Elwert et al. 2010; Gasco et al. 2015; Stadtlander et al. 2017; Wang and Shelomi 2017; Belghit et al. 2019). Most recent studies have focused on the last larval instar and the pupa, since these stages contain the maximum amount of substrate ingested by earlier instars (e.g., Miranda et al. 2019; Ferdousi et al. 2020; Permana et al. 2021). Surprisingly, there is no general consensus on the number of larval stages yet, although larvae of *H. illucens* are also used in forensic entomology as an indicator of the minimum postmortem interval (e.g., Pujol‐Luz et al. 2008; Martínez‐Sánchez et al. 2011). Works on the larval development by May (1961) and Schremmer (1986) reported six and seven larval instars, respectively, and even recent publications state that larval development proceeds through five (Gerhardt and Hribar 2019), six (Kim et al. 2010; Barros et al. 2019a, 2019b; Bruno et al. 2020) or seven (Gligorescu et al. 2019) larval stages.

Since the morphological work of Schremmer (1986) on the penultimate and final instar of *H. illucens*, it is known that the last larval molt is accompanied by a profound change in the head morphology and also lifestyle. Moreover, as it is typical for members of the Stratiomyidae, the pupation takes place within the skin of the last instar, resulting in the formation of a puparium. Hence, what was interpreted by some authors as the pupa (Kim et al. 2010; Barros et al. 2019b; Bruno et al. 2020) is in fact the skin of the last larval instar. This misinterpretation of the ontogeny of *H. illucens* is likely the reason that recent studies focusing on the larval head morphology have not covered the extensive structural changes between the penultimate and ultimate instars adequately. Barros et al. (2019b) examined the external morphology of different larval stages in detail, but the documentation of the mouthparts was limited. Later, Bruno et al. (2020) carried out an in‐depth morpho‐functional analysis of the mouthparts of the penultimate instar. However, a detailed morphological description of the last larval stage was still lacking. In our study, we cover the entire series of stages but mainly focus on the transformation of cephalic structures of the penultimate and the last larval instar. Different structural complexes are described in detail for the first time, such as the drain channels of the digestive system, metacephalic rods, and the cephalic musculature. Functional aspects of morphological differences between the last two larval instars are discussed, with respect to distinct changes in the larval behavior and habits. Even though the number of samples of some of the stages was not sufficient for an unambiguous clarification of the number of larval instars, we provide relevant data on the length of the head capsule and body of all larvae at our disposal.

## 2 Material and Methods

### 2.1 Material

Larvae of *Hermetia illucens* were obtained from a culture of the Hermetia AG (Baruth, Germany). To ensure optimal anatomical results, exemplars of all stages were fixed in FAE (formaldehyde‐ethanol‐acetic acid 3:6:1) or in Bouin (picric acid, acetic acid, formaldehyde). Larvae used for measurements were fixed in 70% ethanol.

### 2.2 Methods

Scanning electron microscopy (SEM) was used to visualize external features. Specimens were dehydrated in an ascending ethanol series (70%–100% ethanol and 99.9% acetone) and dried at the critical point (EmiTech K850 Critical point Dryer, Quorum Technologies Ltd., Ashford, Kent, UK). They were glued on a fine pin, sputter coated with gold (EmiTech K500 sputter coater, Quorum Technologies Ltd., Ashford, Kent, UK), and mounted on a rotatable specimen holder (Pohl 2010). Images were taken with a Philips XL 30 ESEM (Philips, Amsterdam, Netherlands) using Scandium software (Olympus, Münster, Germany).

Histological sections were used to investigate internal structures. The specimens were fixed in FAE (formaldehyde‐ethanol‐acetic acid 3:6:1) and dehydrated with ethanol (80%–100% ethanol) and acetone (99.9%). They were embedded in Araldite (CY 212, Agar Scientific, Stansted/Essex, UK), sectioned (1 µm) with a diamond knife (Elementsix) on a microtome (HM 360, Microm, Waldorf, Germany) and stained with Toluidine blue and Pyronin G (Waldeck GmbH and Co. KG/Division Chroma, Münster, Germany). The sections were digitalized with a Zeiss Axioscope (Carl Zeiss AG, Jena, Germany) with a PixeLINK PL‐B686 digital camera, using the software PixeLINK Capture OEM 7.12 (PixeLINK, Ottawa, Canada). The alignment of the image stack was calculated by Amira® 5.3 (Visage Imaging GmbH, Berlin, Germany) software.

Micro‐Computer‐Tomography (µCT) was used to document external and especially internal structures. Specimens were dehydrated with an ascending ethanol series (70%–100% ethanol) and acetone (99.9%), dried at the critical point (EmiTech K380 Critical point Dryer, Quorum technologies Ltd., Ashford, Kent, UK) and mounted on a metal rod with super glue. The scans were performed with a phoenix/x‐ray nanotom (GE Inspection Technologies, Ahrensburg, Germany) at DESY (Deutsches‐Elektronen‐Synchrotron, Hamburg) using a photon energy of 70 kV.

Computer based three‐dimensional reconstruction: The arrangement of external and internal structures was visualized with a 3D‐reconstruction, based on digitalized and aligned section series. The image stacks of the head were reconstructed with AMIRA® 5.3 (Visage Imaging GmbH, Berlin, Germany) software. The surfaces were polished, smoothed and rendered with MAYA® 2015 (Autodesk, San Rafael, USA).

Terminology: The musculature was named according to the terminology of Kéler (1963) for all muscles that could be clearly homologized.

Measurement of the larvae: For the classification of the larval stages, the length of the head capsule and the total length were measured with an object micrometer on a Zeiss Stemi SV 11 (Carl Zeiss AG, Jena, Germany), in total 837 specimens (L1 = 10, L2 = 202, L3 = 189, L4 = 222, L5 = 177, L6 = 24, L7 = 13). Only larvae preserved in 70% ethanol were used to exclude inaccuracies resulting from artefacts caused by different fixation methods.

Figure Plates: To compile and label figure plates, Adobe Photoshop® version 24.5.0 (Adobe, San José, USA) and Adobe Illustrator® version 27.6.1 (Adobe, San José, USA) were used. The scatterplot showing the distribution of body and head capsule length of measured larvae was created by using the “ggplot2” package (Wickham 2016) in RStudio version 2024.12.1 build 563 (R Studio Team 2016).

## 3 Results

### 3.1 Head Capsule

**FIGURE 1:**
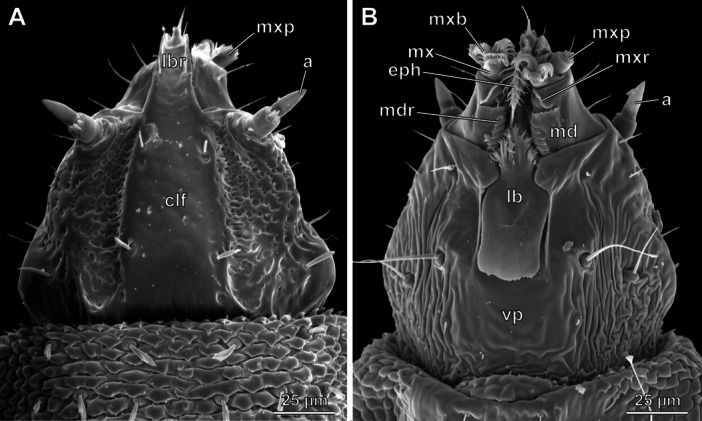
*Hermetia illucens*, head of 1st instar, SEM images. (A) dorsal view. (B) ventral view. Abbreviations: a – antenna, clf – clypeofrontal region, eph – epipharynx, lb – labium, lbr – labrum, md – mandibular part of the mandibulo‐maxillary‐complex, mdr – mandibular ridges, mx – maxillary part of the mandibulo‐maxillary‐complex, mxb – maxillary brush, mxp – maxillary palp, mxr – maxillary ridge, vp – ventral plate.

**FIGURE 2:**
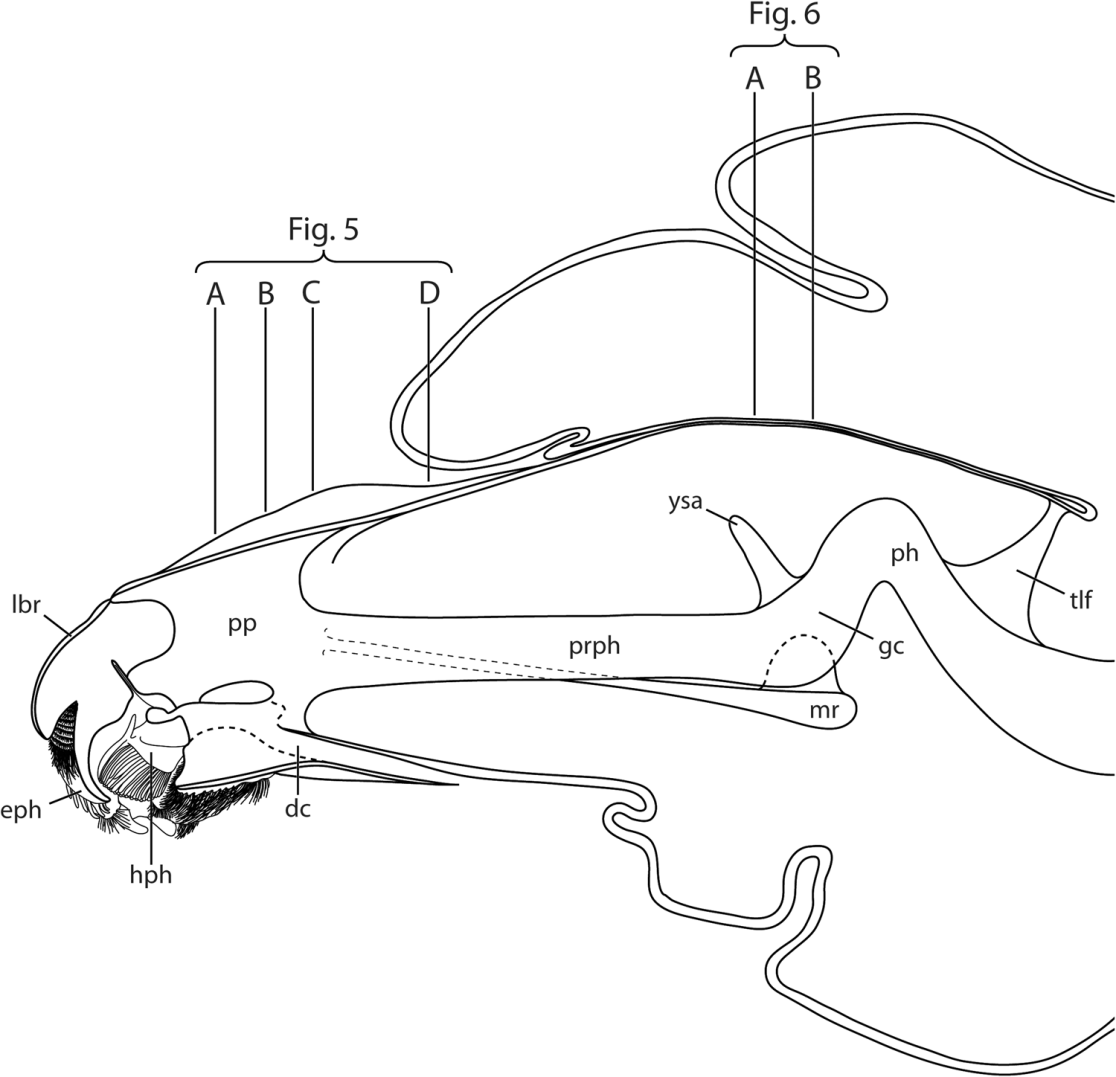
*Hermetia illucens*, internal head capsule, slightly schematized based on confocal microscopy images of the penultimate instar. Mesal view. Orientation: ← anterior, ↓ ventral. Planes of cross sections in Figures 5 and 6 are indicated. Abbreviations: dc – drain channel, eph – epipharynx, gc – grinding chamber, hph – hypopharynx, lbr – labrum, mr – metacephalic rod, ph – pharynx, pp – paraclypeal phragma, prph – prepharynx, tlf – tendon‐like filament, ysa – Y‐shaped apodeme.

The distinctly sclerotized prognathous head of the 1st instar is well developed, partly exposed in its normal position and retractable (Figure 1, Figures [Link], [Link], [Link], [Link]). It is roughly triangular in dorsal view, distinctly narrowing anteriorly, slightly rounded anterolaterally and somewhat bulging posterolaterally. Frons and clypeus are completely fused, thus forming a solid clypeofrontal sclerite (frontoclypeal apotome), without a trace of a clypeofrontal strengthening ridge (Figure 1A: clf). The antennae are inserted anterolaterally on a moderately prominent ring‐shaped socket and a distinct articulatory membrane (Figure 1A: a). The ventral side of the head capsule is closed by an indistinctly delimited, roughly quadrangular sclerotized and smooth ventral plate, possibly of labial origin (Figure 1B: vp). This structure nearly reaches the posteroventral margin of the head. Internally, the extensive paraclypeal phragmata extend dorsoventrally through the anterior head capsule (Figure 2: pp). They divide the anterior head in one dorsomedial and two lateral compartments and thus reinforce the head capsule. Posteriorly, the paraclypeal phragmata merge with the cibarium without a recognizable boundary between both structures. In this transition zone, rod‐shaped sclerites arise laterad the anterior cibarium and extend posteriorly into the thorax (Figure 2: mr). These metacephalic rods are mostly cylindrical but flattened posteriorly in a spoon‐like fashion. They have a concave surface that faces ventrad and serves as a muscle attachment area. At their posterior end, the metacephalic rods are suspended from the posterior end of the cibarium. Posterodorsally, the head capsule is distinctly elongated, reaching far into the thorax. From about half length of the entire head capsule, its cuticle is fused with that of the prothorax, forming a double layer enclosing smaller cavities, especially laterally and posteriorly (Figure 2). This extensive concealed occipital region serves as attachment site for pharyngeal and mandibular muscles.

**FIGURE 3:**
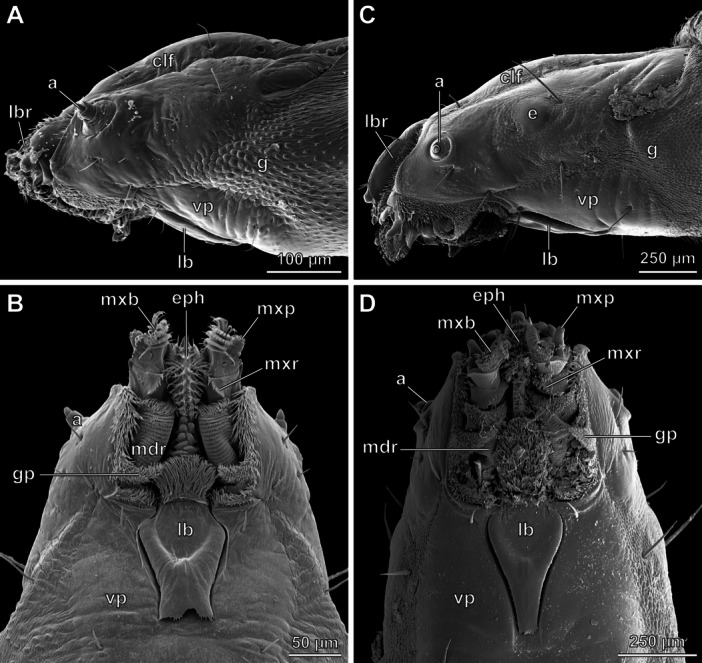
*Hermetia illucens*, head of 3rd and penultimate instar, SEM images. (A) 3rd instar, lateral view. (B) 3rd instar, ventral view. (C) penultimate instar, lateral view. (D) penultimate instar, ventral view. Abbreviations: a – antenna, clf – clypeofrontal region, e – eye, eph – epipharynx, g – gena, gp – triangular genal projection, lb – labium, lbr – labrum, mdr – mandibular ridges, mxb – maxillary brush, mxp – maxillary palp, mxr – maxillary ridge, vp – ventral plate.

The general appearance of the head capsule from instar 2 to the penultimate instar is similar. Notable differences include the eyes, which are located on the lateral cephalic region. The eyes of the first to the penultimate instar consist of a single stemma and become more prominent in later instars (compare Figure 3A, C: e). From the 3rd instar onwards, the genae bear a triangular, medioventrally oriented projection on their ventral margin which is densely covered with setae (Figure 3B, D: gp). The distal portion of the metacephalic rods of instar 6 acquires a distinctly thickened, club‐shaped form before flattening in a spoon‐like manner. It is much more pronounced than in the previous instars.

**FIGURE 4:**
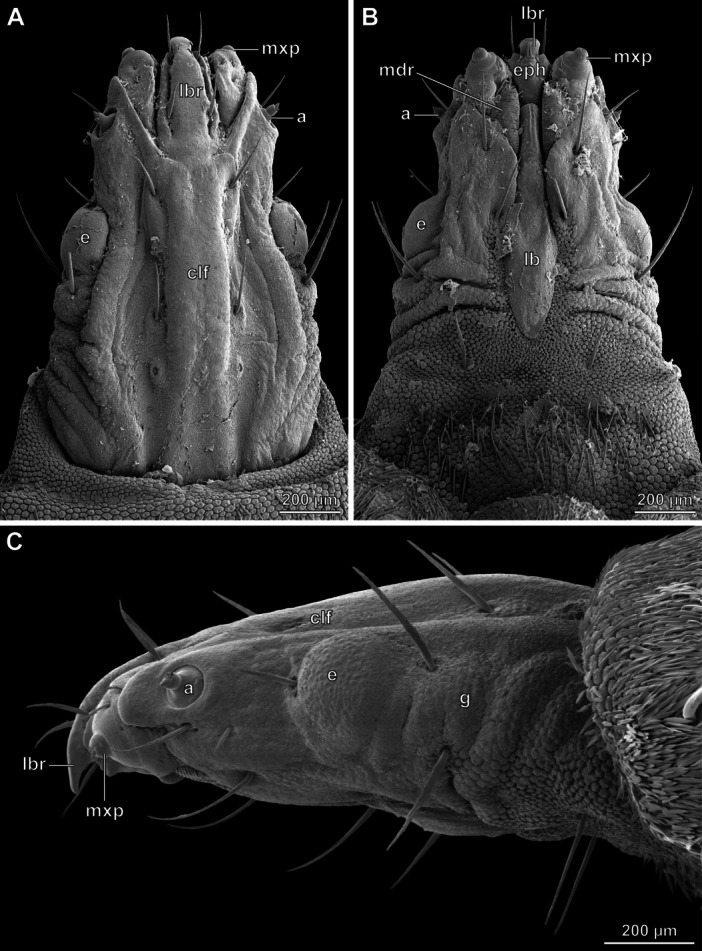
*Hermetia illucens*, head of the ultimate instar, SEM images. (A) dorsal view. (B) ventral view. (C) lateral view. Abbreviations: a – antenna, clf – clypeofrontal region, e – eye, eph – epipharynx, g – gena, lb – labium, lbr – labrum, mdr – mandibular ridges, mxp – maxillary palp.

The head capsule of the last larval instar differs distinctly from that of the earlier larval stages (Figure 4). The clypeofrontal region is narrower and distinctly delimited by a change in the surface texture (Figure 4A). In ventral view, the triangular projection of the genae is missing (Figure 4B). The metacephalic rods are thin along their entire length. In contrast to the previous instars, the ultimate instar possesses compound eyes that are located on a distinct bulge (Figure 4: e).

### 3.2 Labrum

The labrum is placed between the mandibulo‐maxillary complex and completely fused with the anterior clypeal region. In the ultimate instar, the distal labral portion is bent downwards in a hook‐like manner and apically pointed (Figure 4C: lbr). In contrast, the tip of the labrum of the other instars is more rounded and slightly bent upwards.

### 3.3 Antenna

The two‐segmented antenna of the 1st instar is inserted laterally, posterior to the joint of the mandibulo‐maxillary complex (Figure 1A: a), on a large circular membranous pad. The proximal antennomere is cylindrical, straight, about as wide as long, and bears five short, pin‐shaped sensilla on its apical surface. The second antennomere is conical and slightly longer than the first.

In relation to the first antennomere, the second gradually decreases in size from the 2nd to the penultimate instar. Four of the five antennal sensilla of these instars are pin‐shaped and one is knob‐shaped and much smaller. The antenna of the last instar is similar to that of the penultimate (Figures 3, 4: a).

### 3.4 Mandibulo‐Maxillary Complex

The mandibles and maxillae are largely fused in the 1st instar, forming a mandibulo‐maxillary complex (Figure 1B). The distal part of the mandible is reduced and the maxilla is strongly modified. The trapezoid basal element of the complex is part of the mandible (Figure 1B: md). It forms the mandibular joint as a small edge on the basolateral border. Its inner surface forms six ledges with posteriorly oriented teeth. A deep incision is present on the inner surface after the anterior third. From the lateral base of this incision, a finely serrated ledge extends anteromesad on the maxillary portion of the complex. The maxillary element is smaller than the mandibular portion (Figure 1B: mx) and carries the cylindrical and one‐segmented maxillary palp (Figure 1B: mxp), as well as three rows of bristles forming a brush‐like structure (Figure 1B: mxb). A tri‐ to tetradentate projection is present dorsomesad the palp.

From instar 2 to the penultimate stage, the general organization of the mandibulo‐maxillary complex is similar to what is found in the 1st instar, but the fine structures become more differentiated. The serrated ledges on the mandibular part and the rows of setae on the maxillary subunit, which form the brush‐like structure, become more numerous after each molt (compare Figures 1, 3). In addition, the apical part of the mandibular region carries a group of setae, which expands during development. The finely serrated ledge on the maxillary portion bears prominent tooth‐like protrusions (Figure 3B, D: mxr). The teeth of the dentate projection dorsomesad the maxillary palp become flattened in a shovel‐like manner from the presumptive instar 4 to the penultimate.

The mandibulo‐maxillary complex of the ultimate instar is distinctly simplified compared to that of the preceding larval stages (Figure 4B). The mandibular part is smaller and the ventral surface is completely covered with rows of small sclerotized tubercles instead of teeth (Figure 4B: mdr). The apical group of mandibular setae is completely reduced. On the maxillary part of the complex, the dentate protrusion and serrated ledge are absent, and also the rows of setae. The palp is dome‐shaped (Figure 4B: mxp) and bears a small field of sensilla on its apical region.

### 3.5 Labium

**FIGURE 5:**
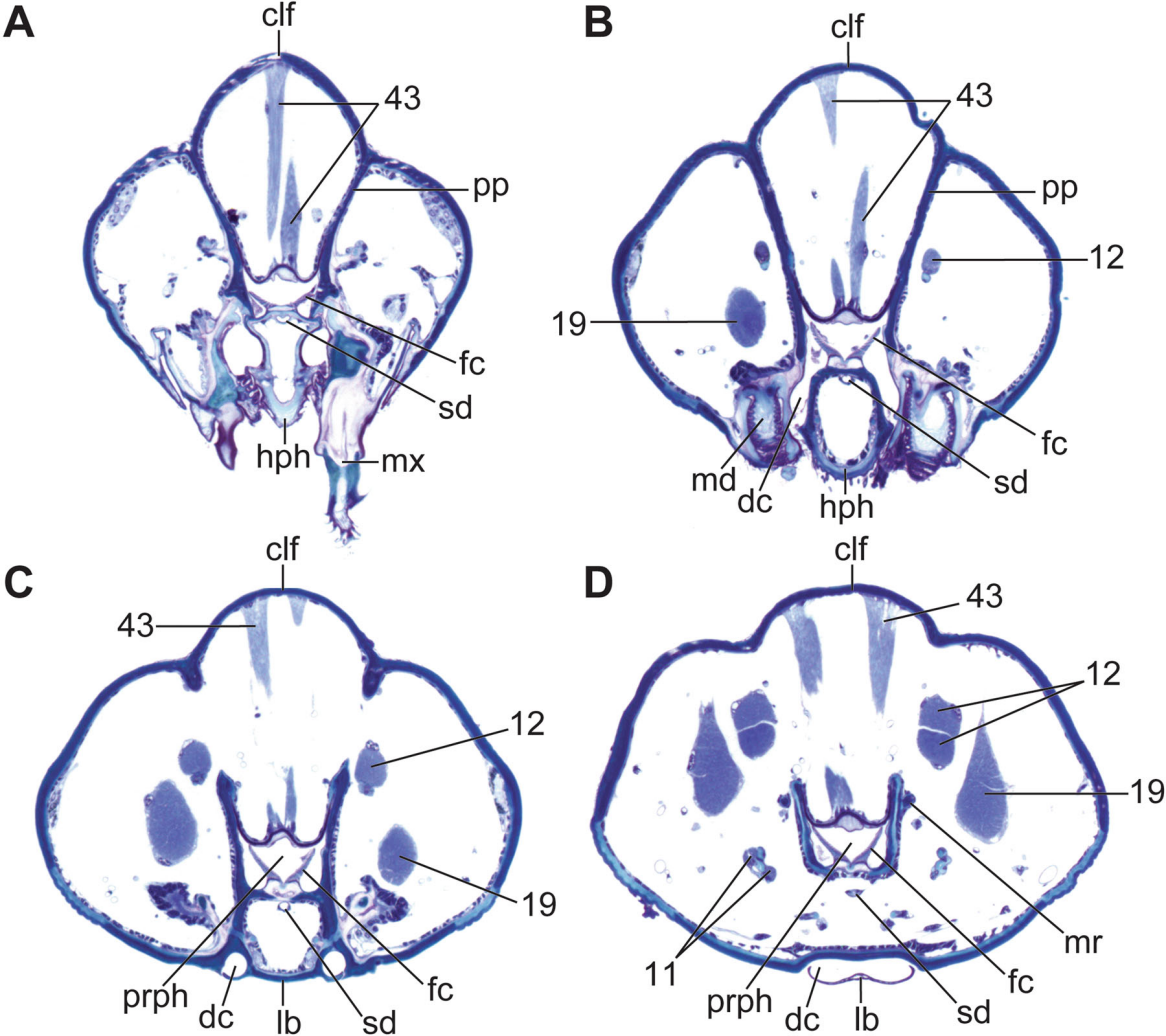
*Hermetia illucens* (3rd instar), cross sections through the anterior part of the head. Orientation: ← lateral, ↓ ventral. The positions of individual planes are indicated in Figure 2. (A, B) sections in the anterior part of the head capsule, showing the moutparts and the paraclypeal fragmata. (C) section posterad the paraclypeal fragmata, showing the fully enclosed prepharynx, filtration combs, drain channels and muscles associated with the mouthparts. (D) section through the middle part of the prepharynx, showing metacephalic rods and the fused drain channels enclosed by the labium. Abbreviations: clf – clypeofrontal region, dc – drain channel, fc – filtration combs, hph – hypopharynx, lb – labium, md – mandibular part of the mandibulo‐maxillary‐complex, mr – metacephalic rod, mx – maxillary part of the mandibulo‐maxillary‐complex, pp – paraclypeal phragma, prph – prepharynx, sd – salivary duct, 11 – M. craniomandibularis internus, 12 – M. craniomandibularis externus, 19 – M. craniolacinialis, 43 – M. clypeopalatalis.

A border between the premental part of the prelabium and the mentum is not recognizable in the 1st instar (Figure 1B: lb). The posterior submental labial part is likely formed by the plate that is visible ventrally. The posterior margin of this structure bears a row of minute teeth. It is relatively thin, of rhomboid shape, appears curved in cross section, and is enclosed in a depression of the head capsule. An indistinct bulge is recognizable ventrally. The plate encloses a cavity that is posteriorly and laterally open and connected with the anterior opening of the cibarium via two lateral drain channels (Figure 5: lb, dc). The bulge that is visible exteriorly marks the point where both channels merge into the large cavity. The anterior prelabial third, likely homologous to the prementum, is tapering anteriorly and bears a group of branched setae. Labial palps are completely missing.

From the 2nd to the penultimate instar, the anterior region enlarges continuously and bears an increasing number of setae. During these instars, the plate gradually becomes more elongated and its posterior margin tapers while the bulge becomes more prominent (compare Figures 1, 3: lb, Figure S2).

In the ultimate instar, the labial plate differs distinctly from that of previous stages. Its overall shape is spoon‐like. The anterior region lacks setae and the bulge in the posterior region is indistinct (Figure 4B: lb). Unlike in previous instars, the posterior region is not placed in a depression of the head capsule.

### 3.6 Epi‐ and Hypopharynx

**FIGURE 6:**
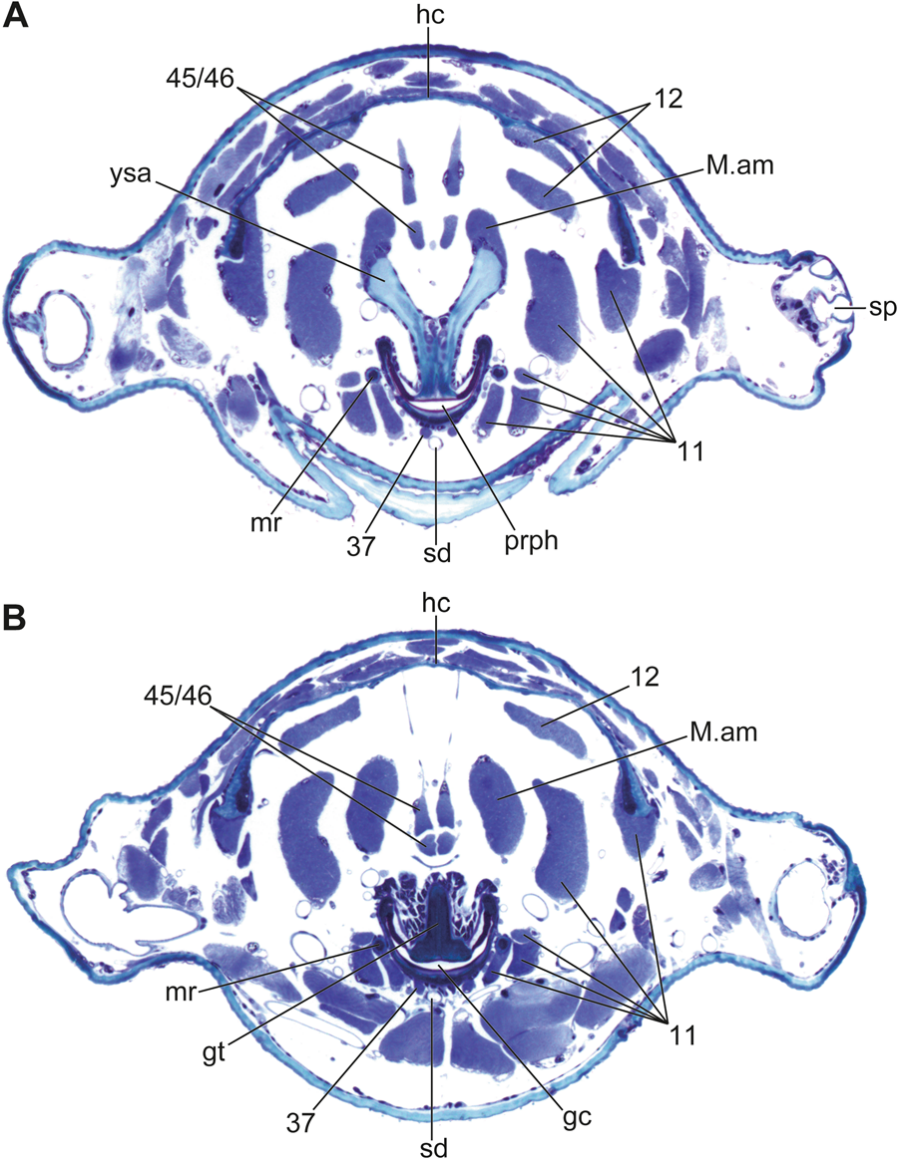
*Hermetia illucens* (3rd instar), cross sections through the posterior part of the head, which extends into the thorax. Orientation: ← lateral, ↓ ventral. The positions of individual planes are indicated in Figure 2. (A) section through the Y‐shaped apodeme. (B) section through the grinding chamber. Abbreviations: gc – grinding chamber, gt – grinding tooth, hc – head capsule, M. am – M. adductor molaris, mr – metacephalic rod, prph – prepharynx, sd – salivary duct, sp – spiracle, ysa – Y‐shaped apodeme, 11 – M. craniomandibularis internus, 12 – M. craniomandibularis externus, 37 – M. hypopharyngosalivarialis, 45/46 – M. frontopharyngalis anterior/posterior.

The exposed anterior epipharynx of the 1st instar covers the ventral side of the labral region and bears a transverse row of paired setae (Figure 1B: eph). The posterior margin of the plate‐like anterior hypopharyngeal part bears two ventral and two dorsal extensions. The salivary duct, unpaired over most of its length (Figure 5: sd), is connected to paired salivary glands posteriorly. It opens on the anterior apex of the hypopharynx and is suspended by a thin muscle (Figure 6: 37), which originates ventrally at the distal end of the metacephalic rods. The epi‐ and hypopharynx are laterally connected by a membrane, thus forming an elongate, U‐shaped, laterally closed cibarium or prepharyngeal tube (Figures 2, 5, 6: prph). It reaches beyond the posterior margin of the head on the ventral side. Anterad the opening of the cibarium, the hypopharynx bears a membrane that fits dorsally in an epipharyngeal slit, thus forming a cibarial closing mechanism. The hypopharyngeal part of the cibarium bears a membranous crest dorsomedially that extends caudad along its entire length. From this crest, two pinnate lamellae extend dorsolaterad to the side walls of the cibarium, separating the cibarial cavity into a dorsomedial and two ventrolateral compartments and forming a filter apparatus (Figure 5: fc). The membranous epipharynx forms the flexible roof of the cibarium and is connected to a strong dilatator muscle, which expands the cibarial volume when it contracts (Figure 5: 43).

The complexity of the anterior epipharyngeal region increases until the penultimate instar, and the number of setae also increases gradually (Figures 1, 3: eph). In addition, the setae are apically split (several times in later instars). Posteriorly, the epipharynx bears a series of plate‐like structures, which become more numerous with each molt. This part is laterally enclosed by the mandibulo‐maxillary complex and posteriorly by the labium.

The epipharyngeal group of setae and the plate‐like structures are completely absent in the last instar (Figure 4B: eph), and the surface of the epipharynx is smooth.

### 3.7 Cephalic Digestive Tract

The anterior part of the cephalic digestive tract is formed by the laterally closed cibarium, that is, the prepharynx, and a complex filter and grinding apparatus. The roof of the long prepharyngeal tube is membranous and formed by the posterior epipharynx (palatum), whereas the solid ventral wall is formed by the posterior hypopharynx. The characteristic grinding chamber (Figures 2, 6: gc) lies below the frontal ganglion, and thus in the region of the anatomical mouth opening in the posterior cephalic region. It is likely formed by the posteriormost part of the cibarium and the anteriormost part of the pharynx. A Y‐shaped apodeme (Figures 2, 6: ysa) serves as attachment area for muscles. Dorsally, both arms of this apodeme broaden. Posteriorly, the apodeme decreases in size and merges with the grinding tooth that is located dorsally in the grinding chamber below the frontal ganglion (Figure 6B: gc, gt). The ventral surface of the grinding tooth appears smooth anteriorly and slightly rough posteriorly. The bowl‐like ventral counterpart of the tooth is characterized by a thickened ventral wall with a smooth surface. Pinnate lamellae of the dorsal side of the hypopharynx almost reach the Y‐shaped apodeme posteriorly. Posterior to the grinding chamber, the pharynx is suspended from the posterior end of the dorsal head capsule by a tendon‐like filament (Figure 2: tlf).

**FIGURE 7:**
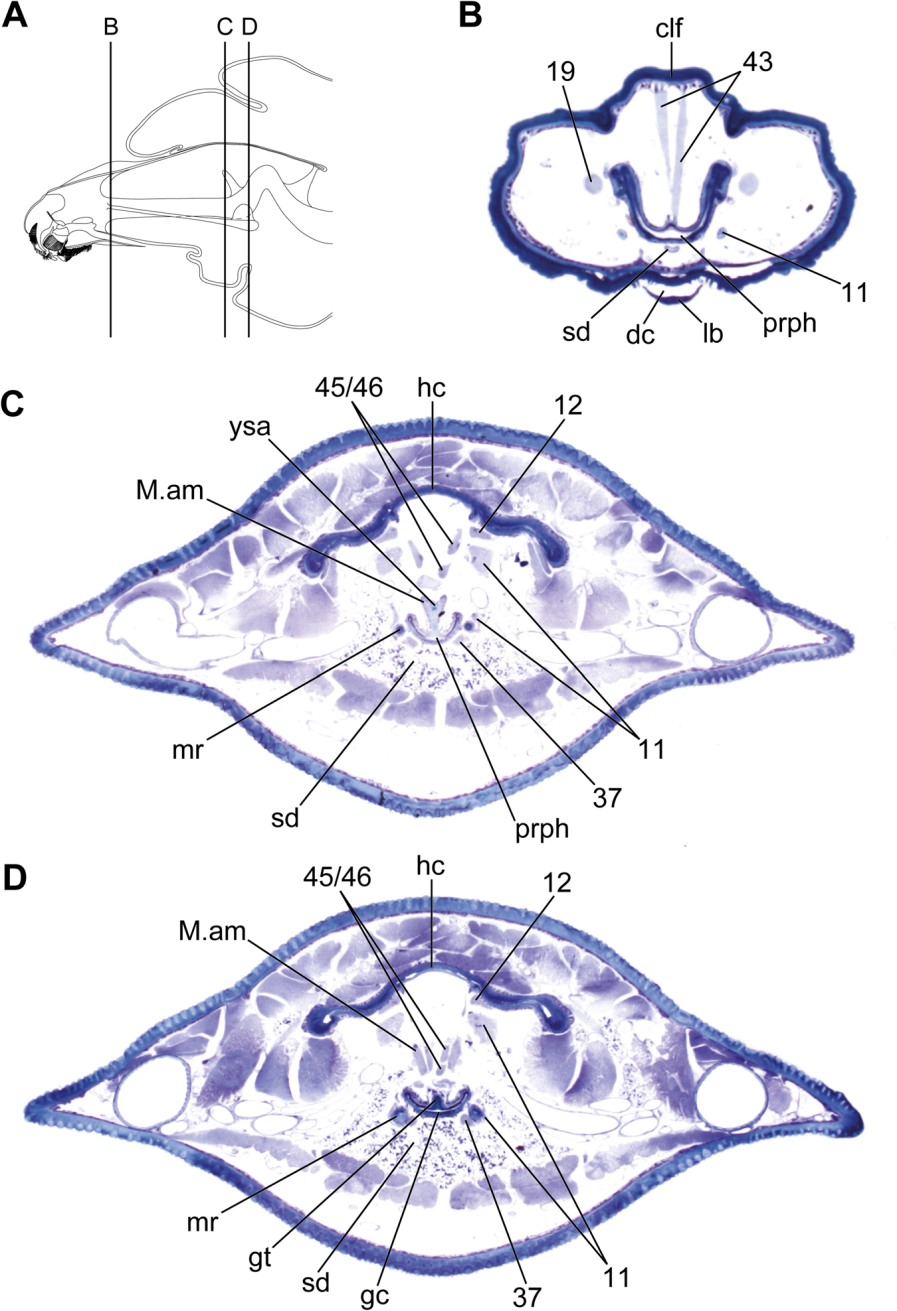
*Hermetia illucens* (ultimate instar), cross sections through the head, which extends into the prothorax in C and D. Orientation: ← lateral, ↓ ventral. (A) indicates the position of sections shown in B–D. Abbreviations: clf – clypeofrontal region, dc = drain channel, gc – grinding chamber, gt – grinding tooth, hc – head capsule, lb – labium, M. am – M. adductor molaris, mr – metacephalic rod, prph – prepharynx, sd – salivary duct, ysa = Y‐shaped apodeme, 11 – M. craniomandibularis internus, 12 – M. craniomandibularis externus, 19 – M. craniolacinialis, 37 – M. hypopharyngosalivarialis, 43 – M. clypeopalatalis, 45/46 – M. frontopharyngalis anterior/posterior.

The structure of the cephalic digestive tract is similar in the earlier and penultimate larval stages, but the pharynx of the last larval instar differs strikingly. The pinnate lamellae of the hypopharynx are absent in the last stage (Figure 7B). Additionally, the Y‐shaped apodeme (Figure 7C: ysa) and the grinding tooth (Figure 7D: gt) are greatly reduced. The ventral wall of the cibarium and the grinding chamber in particular are much thinner and the diameter of the entire digestive tract is considerably smaller.

### 3.8 Musculature

**FIGURE 8:**
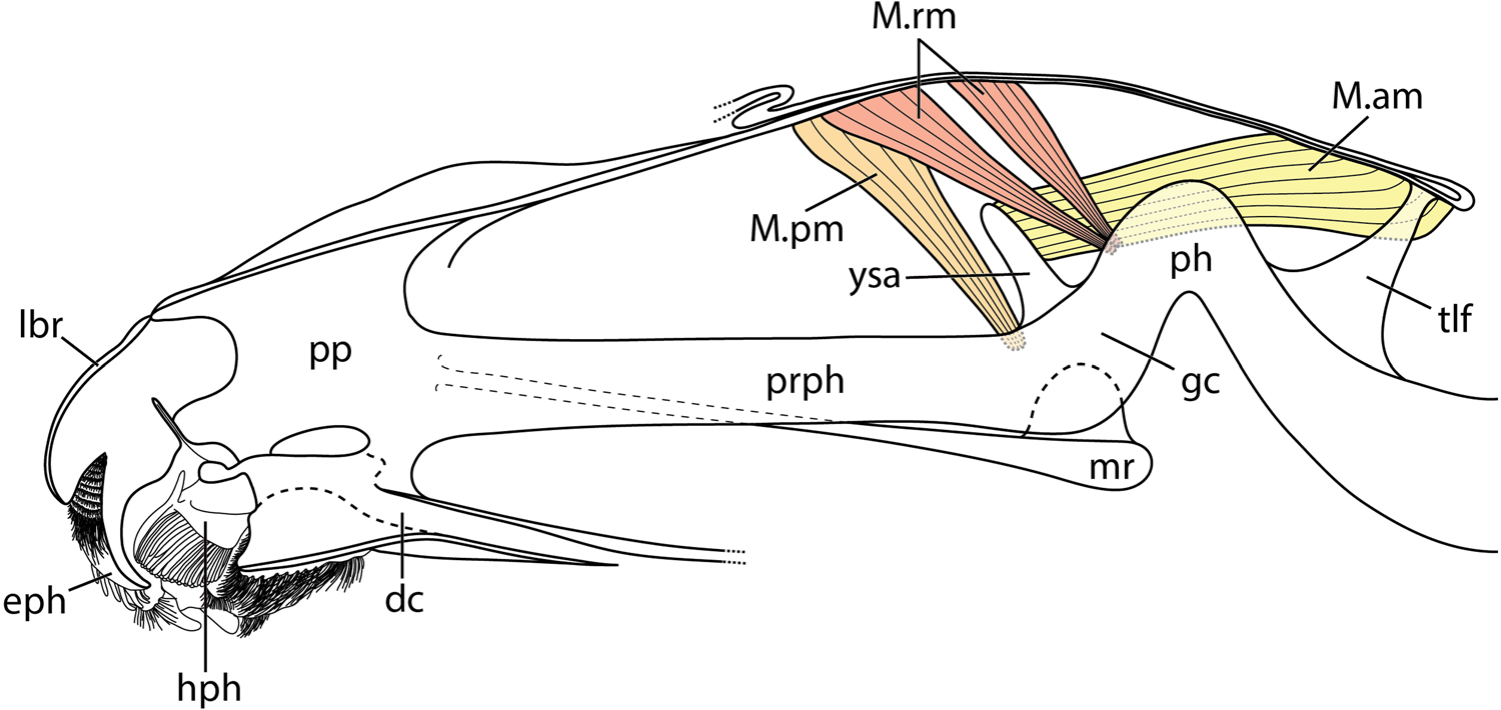
*Hermetia illucens*, internal head capsule and muscles that are associated with the grinding chamber and the grinding tooth, slightly schematized based on confocal microscopy images of the penultimate instar. Mesal view. Orientation: ← anterior, ↓ ventral. Abbreviations: dc – drain channel, eph – epipharynx, gc – grinding chamber, hph – hypopharynx, lbr – labrum, M. am – Musculus adductor molaris, M. pm – Musculus protractor molaris, mr – metacephalic rod, M. rm = Musculus retractor molaris, ph – pharynx, pp – paraclypeal phragma, prph = prepharynx, tlf – tendon‐like filament, ysa – Y‐shaped apodeme.

**FIGURE 9:**
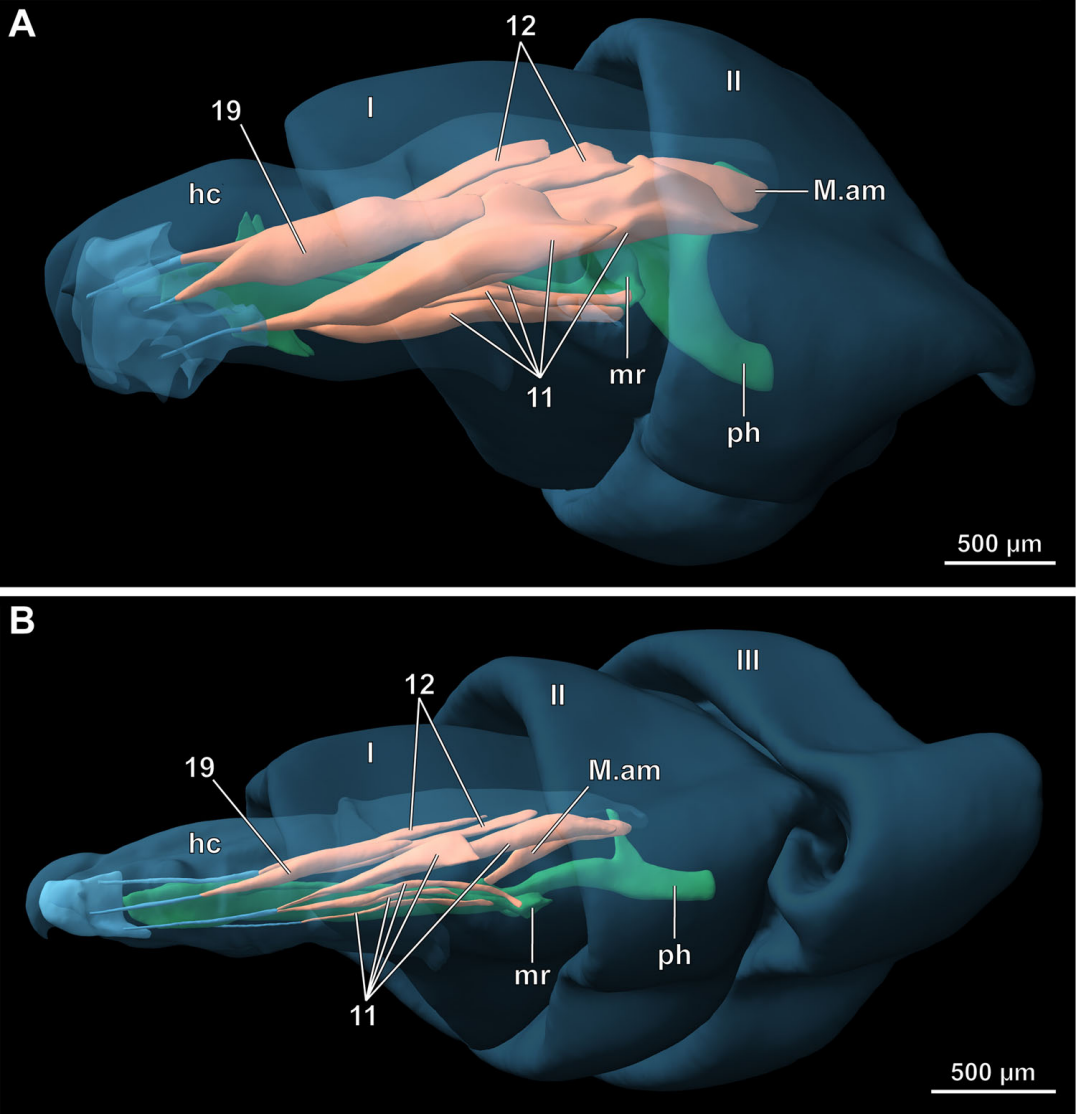
*Hermetia illucens*, renders of 3D reconstructions based on µCT data, lateral view. Orientation: ← anterior, ↓ ventral. (A) penultimate instar. (B) ultimate instar. Abbreviations: hc – head capsule, I – prothorax, II – mesothorax, III – metathorax, M. am – M. adductor molaris, mr – metacephalic rod, ph – pharynx, 11 – M. craniomandibularis internus, 12 – M. craniomandibularis externus, 19 – M. craniolacinialis. Colors: blue – cuticle and mandibulo‐maxillary‐complex with tendons of musculature, green – digestive tract and metacephalic rod, orange – musculature.

The cephalic musculature (Table 1), which includes unique muscles associated with the grinding tooth of the grinding chamber (Figure 8), is the same in all larval stages. However, the specific condition of individual muscles differs distinctly in the last instar. In the last larval stage, muscles that are associated with the cephalic digestive tract and mandibulo‐maxillary complex are only weakly developed (Figure 9) compared to their equivalents in the earlier instars.

**TABLE 1:** Musculature of the head capsule.

Muscle No. (Kéler 1963)	Muscle name	Origin	Insertion	Function
11	M. craniomandibularis internus	2 areas on the lateral head capsule, 3 areas on the metacephalic rods (2 dorsolaterally, 1 ventrally)	With a long tendon on the anteromesal edge of the mandibular part of the complex	Adductor of the mandible
12	M. craniomandibularis externus	Dorsal wall of the head capsule, laterad the pharyngeal dilators	With a long tendon on the dorsal edge of the mandibular part of the complex	Abductor of the mandibulo‐maxillary complex
19	M. craniolacinialis	Lateral wall of head capsule, laterad M. 12	Apical region of the maxillary part of the complex, mesad the maxillary palp	Retractor of the maxillary part
37	M. hypopharyngo‐salivarialis	Posteroventral surface of the metacephalic rods	Dorsal side of the salivary duct	Dilator of the salivarium
43	M. clypeopalatalis	Anterodorsal wall of the head capsule	Roof of the cibarium	Dilator of the cibarium
44	M. clypeobuccalis	Dorsal wall of the head capsule, posterad M. 43	Roof of the cibarium, anterad the frontal ganglion	Dilator of the cibarium
45	M. frontopharyngalis anterior	Posterior dorsal wall of the head capsule	Dorsal folds of the pharynx, posterad the frontal ganglion	Dilator of the pharynx
46	M. frontopharyngalis posterior	Absent or completely fused with M. 45		
68	M. annularis stomodaei	Ring muscle layer around the pharynx		Peristaltic movement of the pharynx
69	M. longitudinalis stomodaei	Longitudinal muscle, along the dorsal pharyngeal wall		
	M. adductor molaris	Dorsal wall of the posterior head capsule	Posterodorsal side of the Y‐shaped apodeme	Adductor of the grinding tooth
	M. protractor molaris	Dorsal wall of the posterior head capsule	Anterior base of the Y‐shaped apodeme	Protractor of the grinding tooth
	M. retractor molaris	Dorsal wall of the posterior head capsule, posterad M. protractor molaris	Grinding tooth	Retractor of the grinding tooth
	Ventral retractor of head capsule	Extensive area on the ventrolateral wall of the posterior prothoracic region	Wide area on the ventral hind margin of the head capsule (ventral plate)	Retractor of the head capsule
	Dorsal retractor of head capsule 1	Dorsomedian wall of prothorax	Median muscle band on the dorsal wall of the head capsule	Retractor and levator of the head capsule
	Dorsal retractor of head capsule 2	Extensive areas of the dorsal wall of the prothorax, posterior to dorsal retractor of the head capsule 1	Wide area on the dorsal wall of the head capsule, laterad the dorsal retractor of the head capsule 1	Retractor of the head capsule

### 3.9 Classification of the Larval Instars

**FIGURE 10:**
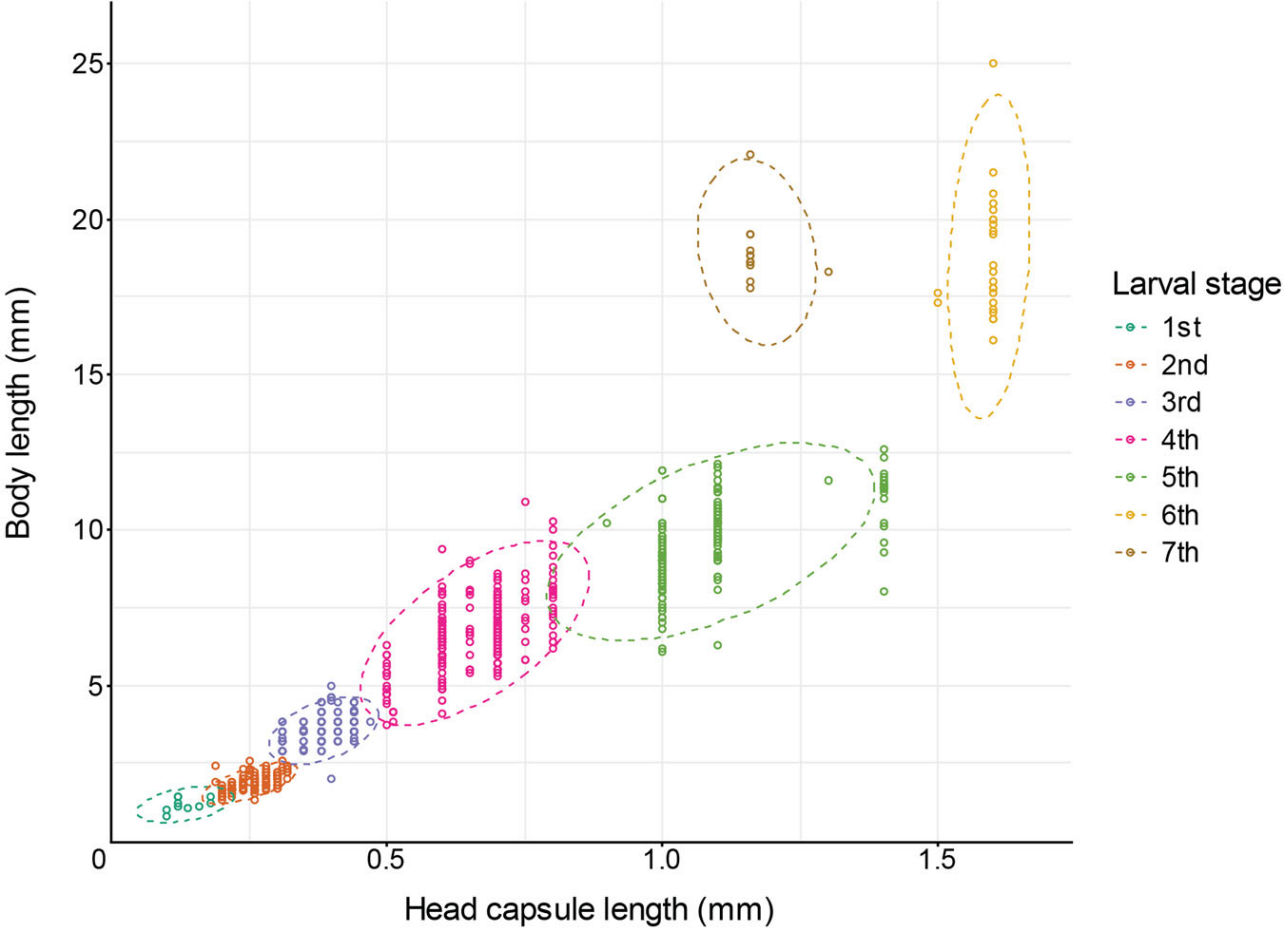
Scatter plot showing the distribution of the body length and head capsule length of all measured *Hermetia illucens* larvae. The assignment to different larval stages is indicated by the colors. All measurements are provided in Supporting Information S1: Data 1.

We measured 837 specimens and tentatively categorized them based on head capsule and body length (Table 2, Figure 10). The body length of the 1st instar ranges from 0.80 mm to 1.40 mm. During the postembryonic development, successive larval instars increase in size until the penultimate instar, at which they can reach a maximum length of up to 25 mm. On average, the ultimate instar is slightly longer than the penultimate, but their maximum length reaches only 22.1 mm. Similar to the body length, the head capsule length increases until the penultimate instar. The head capsule of the last instar is shorter than that of the penultimate and only slightly longer than that of the previous instar. There was a strong overlap of body lengths between the presumptive first 4th and 5th instars, and the penultimate and last instar. However, both can be distinguished by considerable differences in head capsule length. Although our sampling is not sufficient for a significant result, the data strongly suggest seven larval instars in the postembryonic development.

**TABLE 2:** Measurements of head capsule length and body length for each larval instar.

larval instar (?)	Mean head capsule length [mm]	± SD	Range of head capsule length [mm]	Mean body length [mm]	± SD	Range of body length [mm]
1st	0.13	± 0.03	0.10–0.18	1.16	± 0.20	0.80–1.40
2nd	0.25	± 0.04	0.19–0.32	1.84	± 0.27	1.30–2.55
3rd	0.39	± 0.04	0.31–0.47	3.54	± 0.44	2.00–5.00
4th	0.66	± 0.08	0.50–0.80	6.69	± 1.21	3.70–10.90
5th	1.09	± 0.12	0.90–1.40	9.61	± 1.29	6.10–12.60
6th	1.59	± 0.03	1.50–1.60	18.79	± 2.00	16.10–25.00
7th	1.17	± 0.04	1.16–1.30	18.93	± 1.07	17.80–22.10

*Note*: 837 specimens were measured (L1 = 10, L2 = 202, L3 = 189, L4 = 222, L5 = 177, L6 = 24, L7 = 13). Suggested larval instars are tentative, due to a limited sample size in some stages. All measurements are provided in Supporting Information S1: Data 1.

Abbreviation: SD, standard deviation.

## 4 Discussion

### 4.1 Functional Interpretation of Morphological Characters

The profound modifications between the last two larval instars of the black soldier fly are associated with a behavioral shift from stationary feeding to increased vagility (Schremmer 1986). Among the most striking developmental changes are those of the mouthparts. The basic composition of the mandibulo‐maxillary complex does not change during the earlier larval instars until the penultimate (this study and Bruno et al. 2020). However, we observed that with each molt, they become larger and more complex. In particular, the density of the rows of maxillary bristles, which form a brush‐like structure, and of the serrated ridges on the mandibular part gradually increases. Therefore, it is conceivable that 1st instar larvae mostly rely on highly liquefied substrate, while older larvae are able to process food particles more intensively before ingesting them in substrates with higher viscosity. This ability may be important as black soldier fly larvae are known to inhabit rapidly changing food substrates (Schremmer 1986), which may often dry out during larval development. In addition, the larval activity itself has been shown to reduce moisture in manure, supporting this hypothesis (Sheppard 1983).

Our observations of the composition of the digestive tract indicate that the feeding process of *H. illucens* is probably similar to that described by Schremmer (1951) for another stratiomyid species. The mandibulo‐maxillary complex dislodges and breaks down food particles, potentially liquefying them before ingestion (Bruno et al. 2020). Feeding occurs in two phases, suction and filtration. During suction, the well‐developed dilator muscles attached to the epipharynx contract and create negative pressure, drawing liquefied food into the digestive tract. In the filtration phase, the elastic wall of the epipharynx exerts pressure on the food substrate, which pushes excess water through pinnate lamellae into ventrolateral compartments. Most of the filtered water presumably exits through paired drainage channels that are enclosed by the labium before they exit on the posteroventral side of the head, while concentrated food moves into the grinding chamber, where a grinding tooth processes it against the ventral surface before ingestion. Since the drainage channels are apparently not completely sealed, a portion of the filtered water likely exits through outlet orifices laterad the anterior prelabium. The setation of the anterior prelabium and the triangular genal projections likely prevent mixing of the already filtered water with liquid containing suspended food particles below the anteroventral region of the head.

The mandibulo‐maxillary complex is profoundly altered in the last larval instar (this study, May 1961; Schremmer 1986; Barros et al. 2019b; Gligorescu et al. 2019; Bruno et al. 2020). We demonstrate that the associated musculature is weakly developed, most likely rendering the apparatus largely immobile and nonfunctional. In addition, we found that the filter combs in the prepharyngeal tube are absent and that the grinding chamber and tooth with its associated musculature are greatly reduced. Therefore, it is likely that feeding plays only a minor role in the last instar. However, the presence of mandibular ridges and weakly developed prepharyngeal dilatator muscles suggests that at least the ingestion of liquid substrates is still possible. This assumption is supported by observations of May (1961), who left the last instar on colored substrate and later found that their digestive tracts displayed a similar coloration, indicating that the larvae had consumed some of the material.

The transition from the penultimate to the ultimate instar larva is accompanied by increased vagility. The last instar has been reported to leave the material they feed on to find a suitable place to pupate (May 1961; Schremmer 1986), although under certain conditions they appear to burrow into the food substrate to pupate right there (Barros et al. 2019a). It is conceivable that leaving a substrate that could dry out quickly or attract other animals that could disturb the processes of pupation and metamorphosis is an advantageous behavioral strategy. In addition to the hooked labrum, the enhanced visual abilities indicated by the laterally convex compound eyes likely increase the efficiency of the migration of the last larval instar.

### 4.2 Number of Larval Instars

In our sampling for length measurements, the first larval stage and the last two larval instars are only represented by a small number of individuals. First instar larvae are likely underrepresented as small size and short duration of the stage make it difficult to detect them in the food substrate, especially if not examined at the optimal time. The last two instars are likely underrepresented because only few larvae had reached these stages when our sampling was scheduled at the supplying company. Additionally, the last two stages were prioritized for other initially planned experiments, such as transcriptome analysis, which required different fixation methods and therefore could not be included in our length measurements.

In previous studies, larval instars were determined based on measurements of the body length (Schremmer 1986) or head capsule width (e.g., May 1961; Barros et al. 2019b; Gligorescu et al. 2019) (supporting online material Table 1). Body length alone is not suitable because it depends on the method of fixation and availability and quality of food during the larval development (e.g., Nguyen et al. 2013; Myers et al. 2014; Harnden and Tomberlin 2016). Measurements of the sclerotized and rigid head capsule are more reliable as they are much less dependent on the nutritional state of the larvae. The head capsule length measured in the present study tentatively indicates that the postembryonic development of *H. illucens* proceeds through seven larval instars plus the pupation, confirming the observations of Schremmer (1986) and Gligorescu et al. (2019). As Gligorescu et al. (2019) noted, publications mentioning less larval instars either missed the 1st instar (May 1961) or misinterpreted the 7th instar as the “pupa” (puparium) (Kim et al. 2010; Barros et al. 2019b; Bruno et al. 2020).

The larval development is accompanied by a gradual growth until the penultimate instar is reached. This can be monitored by measuring the width and length of the head capsule or the length of the body. In contrast, the final molt from the penultimate to the ultimate instar is characterized by a decrease in head capsule size (our data; Kim et al. 2010; Barros et al. 2019b; Gligorescu et al. 2019), while the body length can either remain unchanged (Schremmer 1986), increase (Barros et al. 2019b) or decrease (McFadden 1967). Misinterpretations of larval stages in previous studies indicate that size measurements alone are not always sufficient to determine larval instars. In particular, larvae that are either exceptionally small or large for their respective stage are problematic. Therefore, it is advisable not to use a single parameter such as either head capsule length or width, or body length, but a combination of them, and also other morphological characteristics. As we show, especially the transition from the penultimate to the last instar is accompanied by distinctive morphological changes, such as transformations of the mouthparts and the shape of the head capsule. Other characteristics are a change in color, increased calcareous incrustation, changes in the arrangement of bristles, and the formation of thoracic fissure lines (Schremmer 1986). Additionally, earlier observations (Schremmer 1986) and our own data show that the mouthparts are comparatively simple in the 1st larval instar, but that their complexity increases in subsequent postembryonic stages. The gradual change in the shape of the labium between instars, as observed in our study, also serves as a useful indicator for identifying the larval stage. These morphological characters and a correct interpretation of larval stages allow for an easier and more precise determination of larvae, which is of particular interest for the use of *H. illucens* in forensic entomology.

## Author Contributions

**Benjamin Fabian:** methodology, conceptualization, investigation, writing – original draft, writing – review and editing. **Katharina Schneeberg:** conceptualization, methodology, investigation, supervision, funding acquisition, writing – original draft. **Stephan Löwe:** methodology, investigation. **René Bauernfeind:** methodology, investigation. **Rolf Georg Beutel:** conceptualization, investigation, supervision, writing – original draft, writing – review and editing.

### 1 Peer Review

The peer review history for this article is available at https://www.webofscience.com/api/gateway/wos/peer-review/10.1002/jmor.70048.

## Supporting information

Supporting Figure 1 ‐ Larval Head Dorsal.

Supporting Figure 2 ‐ Larval Head Ventral.

Supporting Figure 3 ‐ Larval Head Lateral.

Supporting Figure 4 ‐ Larval Head Frontal.

Supporting Data 1 ‐ Larval measurements.

supmat.

## Data Availability

The data that support the findings of this study are available on request from the corresponding author. The data are not publicly available due to privacy or ethical restrictions.

## References

[jmor70048-bib-0001] Banks, I. J. 2014. To Assess the Impact of Black Soldier Fly (*Hermetia illucens*) Larvae on Faecal Reduction in Pit Latrines. PhD Thesis, London School of Hygiene & Tropical Medicine. 10.17037/PUBS.01917781.

[jmor70048-bib-0002] Barros, L. M. , R. L. Ferreira‐Keppler , R. T. Martins , and A. L. N. Gutjahr . 2019a. “Bionomy of *Hermetia illucens* (Diptera: Stratiomyidae) on Decomposing Swine Carcass in an Urban Area of Central Amazon.” Journal of Medical Entomology 56, no. 3: 681–689. 10.1093/jme/tjz005. 30759224

[jmor70048-bib-0003] Barros, L. M. , A. L. N. Gutjahr , and R. L. F. Ferreira‐Keppler . 2019b. “Morphological Description of the Immature Stages of *Hermetia illucens* (Linnaeus, 1758) (Diptera: Stratiomyiidae).” Microscopy Research and Technique 82: 178–189. 10.1002/jemt.23127. 30511417

[jmor70048-bib-0004] Belghit, I. , N. S. Liland , P. Gjesdal , et al. 2019. “Black Soldier Fly Larvae Meal Can Replace Fish Meal in Diets of Sea‐Water Phase Atlantic Salmon (*Salmo salar*).” Aquaculture 503: 609–619. 10.1016/j.aquaculture.2018.12.032.

[jmor70048-bib-0005] Bondari, K. , and D. C. Sheppard . 1987. “Soldier Fly, *Hermetia illucens* L., Larvae as Feed for Channel Catfish, *Ictalurus punctatus* (Rafinesque), and Blue Tilapia, *Oreochromis aureus* (Steindachner).” Aquaculture Research 18, no. 3: 209–220. 10.1111/j.1365-2109.1987.tb00141.x.

[jmor70048-bib-0006] Bruno, D. , T. Bonacci , M. Reguzzoni , et al. 2020. “An In‐Depth Description of Head Morphology and Mouthparts in Larvae of the Black Soldier Fly *Hermetia illucens* .” Arthropod Structure & Development 58: 100969. 10.1016/j.asd.2020.100969. 32769052

[jmor70048-bib-0007] Elwert, C. , I. Knips , and P. Katz . 2010. “A Novel Protein Source: Maggot Meal of the Black Soldier Fly (*Hermetia illucens*) in Broiler Feed.” In 11. Tagung Schweine‐ und Geflügelernährung, 23.‐25. November 2010, edited by M. Gierus , H. Kluth , M. Bulang , and H. Kluge . Lutherstadt Wittenberg, Institut für Agrar‐ und Ernährungswissenschaften, Universität Halle‐Wittenberg. https://www.feedtest.de/Publikationen/2010%20SGE%20Hermetia%20meal.pdf.

[jmor70048-bib-0008] Ferdousi, L. , N. Sultana , M. A. Helal , and N. Momtaz . 2021. “Molecular Identification and Life Cycle of Black Soldier Fly (*Hermetia illucens*) in Laboratory.” Bangladesh Journal of Zoology 48, no. 2: 429–440. 10.3329/bjz.v48i2.52381.

[jmor70048-bib-0009] Furman, D. P. , R. D. Young , and P. E. Catts . 1959. “ *Hermetia illucens* (Linnaeus) as a Factor in the Natural Control of *Musca domestica* Linnaeus.” Journal of Economic Entomology 52: 917–921. 10.1093/jee/52.5.917.

[jmor70048-bib-0010] Gasco, L. , M. Stas , A. Schiavone , et al. 2015. Use of Black Soldier Fly (*Hermetia illucens*) Meal in Rainbow Trout (*Onchorhynchus mykiss*) feeds. Rotterdam, Netherlands: Aquaculture Europe.

[jmor70048-bib-0011] Gerhardt, R. R. , and L. J. Hribar . 2019. “Flies (Diptera).” In Medical and Veterinary Entomology, edited by G. Mullen and L. Durden , 2002, 171–190. San Diego, California: Academic Press.

[jmor70048-bib-0012] Gligorescu, A. , S. Toft , H. Hauggaard‐Nielsen , J. A. Axelsen , and S. A. Nielsen . 2019. “Development, Growth and Metabolic Rate of *Hermetia illucens* Larvae.” Journal of Applied Entomology 143, no. 8: 875–881. 10.1111/jen.12653.

[jmor70048-bib-0013] Gold, M. , J. K. Tomberlin , S. Diener , C. Zurbrügg , and A. Mathys . 2018. “Decomposition of Biowaste Macronutrients, Microbes, and Chemicals in Black Soldier Fly Larval Treatment: A Review.” Waste Management 82: 302–318. 10.1016/j.wasman.2018.10.022. 30509593

[jmor70048-bib-0014] Harnden, L. M. , and J. K. Tomberlin . 2016. “Effects of Temperature and Diet on Black Soldier Fly *Hermetia illucens* (L.) (Diptera: Stratiomyidae), Development.” Forensic Science International 266: 109–116. 10.1016/j.forsciint.2016.05.007. 27236368

[jmor70048-bib-0015] Kéler, S. v. 1963. Entomologisches Wörterbuch, mit besonderer Berücksichtigung der morphologischen Terminologie. Berlin: Akademie‐Verlag.

[jmor70048-bib-0016] Kim, W. T. , S. W. Bae , H. C. Park , et al. 2010. “The Larval Age and Mouth Morphology of the Black Soldier Fly, *Hermetia illucens* (Diptera: Stratiomyidae).” International Journal of Industrial Entomology 21, no. 2: 185–187.

[jmor70048-bib-0017] Lalander, C. , S. Diener , M. E. Magri , C. Zurbrügg , A. Lindström , and B. Vinnerås . 2013. “Faecal Sludge Management With the Larvae of the Black Soldier Fly (*Hermetia illucens*)—From a Hygiene Aspect.” Science of the Total Environment 458–460: 312–318. 10.1016/j.scitotenv.2013.04.033. 23669577

[jmor70048-bib-0018] Lardé, G. 1990. “Recycling of Coffee Pulp by *Hermetia illucens* (Diptera: Stratiomyidae) Larvae.” Biological Wastes 33, no. 4: 307–310. 10.1016/0269-7483(90)90134-E.

[jmor70048-bib-0020] Lord, W. , M. Goff , T. Adkins , and N. Haskell . 1994. “The Black Soldier Fly *Hermetia illucens* (Diptera: Stratiomyidae) as a Potential Measure of Human Postmortem Interval: Observations and Case Histories.” Journal of Forensic Sciences 39, no. 1: 215–222. 10.1520/JFS13587J. 8113702

[jmor70048-bib-0021] Martínez‐Sánchez, A. , C. Magaña , M. Saloña , and S. Rojo . 2011. “First Record of *Hermetia illucens* (Diptera: Stratiomyidae) on Human Corpses in Iberian Peninsula.” Forensic Science International 206, no. 1–3: e76–e78. 10.1016/j.forsciint.2010.10.021. 21145189

[jmor70048-bib-0022] May, B. M. 1961. “The Occurrence in New Zealand and the Life‐History of the Soldier Fly *Hermetia illucens* (L.) (Diptera: Stratiomyidae).” New Zealand Journal of Science 4, no. 5: 55–65.

[jmor70048-bib-0023] McFadden, M. W. 1967. “Soldier Fly Larvae in America North of Mexico.” Proceedings of the United States National Museum 121, no. 3569: 1–72.

[jmor70048-bib-0024] Mertenat, A. , S. Diener , and C. Zurbrügg . 2019. “Black Soldier Fly Biowaste Treatment–Assessment of Global Warming Potential.” Waste Management 84: 173–181. 10.1016/j.wasman.2018.11.040. 30691890

[jmor70048-bib-0025] Miranda, C. D. , J. A. Cammack , and J. K. Tomberlin . 2019. “Life‐History Traits of the Black Soldier Fly, *Hermetia illucens* (L.) (Diptera: Stratiomyidae), Reared on Three Manure Types.” Animals: An Open Access Journal from MDPI 9, no. 5: 281. 10.3390/ani9050281. 31130651 PMC6563101

[jmor70048-bib-0026] Myers, H. M. , J. K. Tomberlin , B. D. Lambert , and D. Kattes . 2008. “Development of Black Soldier Fly (Diptera: Stratiomyidae) Larvae Fed Dairy Manure.” Environmental Entomology 37, no. 1: 11–15. 10.1093/ee/37.1.11. 18348791

[jmor70048-bib-0027] Newton, G. L. , C. V. Booram , R. W. Barker , and O. M. Hale . 1977. “Dried *Hermetia illucens* Larvae Meal as a Supplement for Swine.” Journal of Animal Science 44: 395–400. 10.2527/jas1977.443395x.

[jmor70048-bib-0028] Nguyen, T. T. X. , J. K. Tomberlin , and S. Vanlaerhoven . 2013. “Influence of Resources on *Hermetia illucens* (Diptera: Stratiomyidae) Larval Development.” Journal of Medical Entomology 50, no. 4: 898–906. 10.1603/ME12260. 23926790

[jmor70048-bib-0029] Permana, A. D. , R. E. Putra , A. Nurulfah , M. Rosmiati , and I. Kinasih . 2021. “Growth of Black Soldier Fly Larvae (*Hermetia illucens*) Fed With Pak Choi (*Brassica chinensis*) and Carp (*Cyprinus carpio*) Residues.” BIOTROPIA 28, no. 2: 92–101. 10.11598/btb.2021.28.2.1078.

[jmor70048-bib-0030] Pohl, H. 2010. “A Scanning Electron Microscopy Specimen Holder for Viewing Different Angles of a Single Specimen.” Microscopy Research and Technique 73: 1073–1076. 10.1002/jemt.2083. 20196104

[jmor70048-bib-0031] Pujol‐Luz, J. R. , P. A. C. Francez , A. Ururahy‐Rodrigues , and R. Constantino . 2008. “The Black Soldier‐Fly, *Hermetia illucens* (Diptera, Stratiomyidae), Used to Estimate the Postmortem Interval in a Case in Amapá State, Brazil*.” Journal of Forensic Sciences 53, no. 2: 476–478. 10.1111/j.1556-4029.2008.00659.x. 18366584

[jmor70048-bib-0032] R Studio Team . 2016. RStudio: Integrated Development for R. Boston, MA: RStudio, Inc. http://www.rstudio.com/.

[jmor70048-bib-0033] Schremmer, F. 1951. “Die Mundteile der Brachycerenlarven und der Kopfbau der Larve von *Stratiomys chamaeleon* L.” Österreichische Zoologische Zeitschrift 3: 326–397.

[jmor70048-bib-0034] Schremmer, F. 1986. “Die Polymetabole Larval‐Entwicklung der Waffenfliegenart *Hermetia illucens*. – Ein Beitrag zur Metamorphose der Stratiomyidae.” Annalen des Naturhistorischen Museums in Wien, Serie B für Botanik und Zoologie 88, no. 89: 405–429.

[jmor70048-bib-0035] Sheppard, C. 1983. “House Fly and Lesser Fly Control Utilizing the Black Soldier Fly in Manure Management Systems for Caged Laying Hens.” Environmental Entomology 12, no. 5: 1439–1442. 10.1093/ee/12.5.1439.

[jmor70048-bib-0036] Stadtlander, T. , A. Stamer , A. Buser , J. Wohlfahrt , F. Leiber , and C. Sandrock . 2017. “ *Hermetia illucens* Meal as Fish Meal Replacement for Rainbow Trout on Farm.” Journal of Insects as Food and Feed 3, no. 3: 165–175. 10.3920/JIFF2016.0056.

[jmor70048-bib-0037] Surendra, K. C. , J. K. Tomberlin , A. van Huis , J. A. Cammack , L. H. L. Heckmann , and S. K. Khanal . 2020. “Rethinking Organic Wastes Bioconversion: Evaluating the Potential of the Black Soldier Fly (*Hermetia illucens* (L.)) (Diptera: Stratiomyidae) (BSF).” Waste Management 117: 58–80. 10.1016/j.wasman.2020.07.050. 32805602

[jmor70048-bib-0038] Wang, Y. S. , and M. Shelomi . 2017. “Review of Black Soldier Fly (*Hermetia illucens*) as Animal Feed and Human Food.” Foods 6, no. 10: 91. 10.3390/foods6100091. 29057841 PMC5664030

[jmor70048-bib-0039] Wickham, H. 2016. Ggplot2: Elegant Graphics for Data Analysis. Springer.

