# Supplementary figures and images for: Transformations of Head Structures During the Larval Development of the Black Soldier Fly Hermetia illucens (Stratiomyidae, Diptera)
Source: J Morphol. 2025 Apr 11;286(4):e70048. doi: 10.1002/jmor.70048 (PMC11992506; doi:10.1002/jmor.70048)

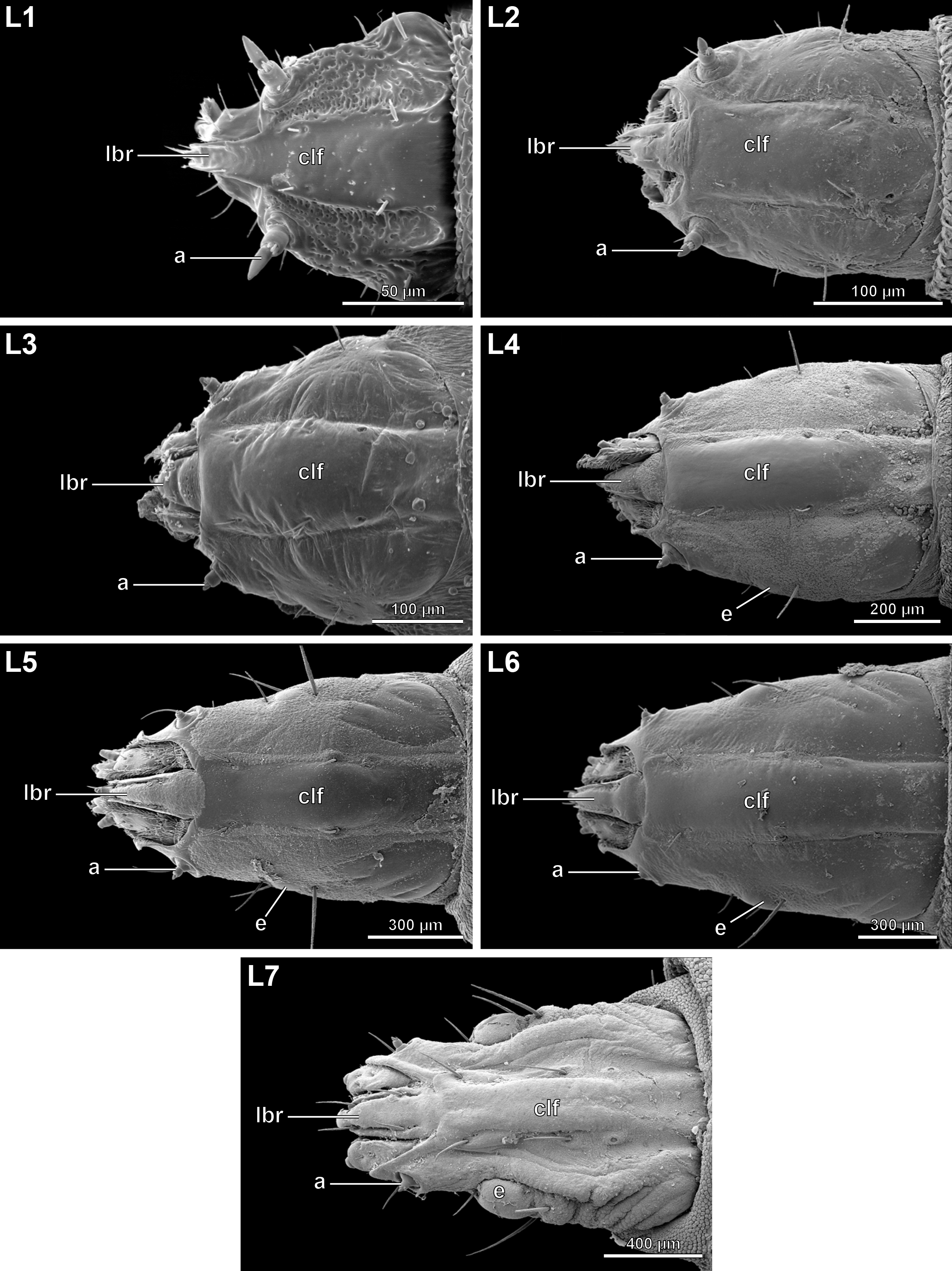

Supplement: Supplementary file 1 — Supporting Figure 1 ‐ Larval Head Dorsal. [file JMOR-286-e70048-s004.jpg]

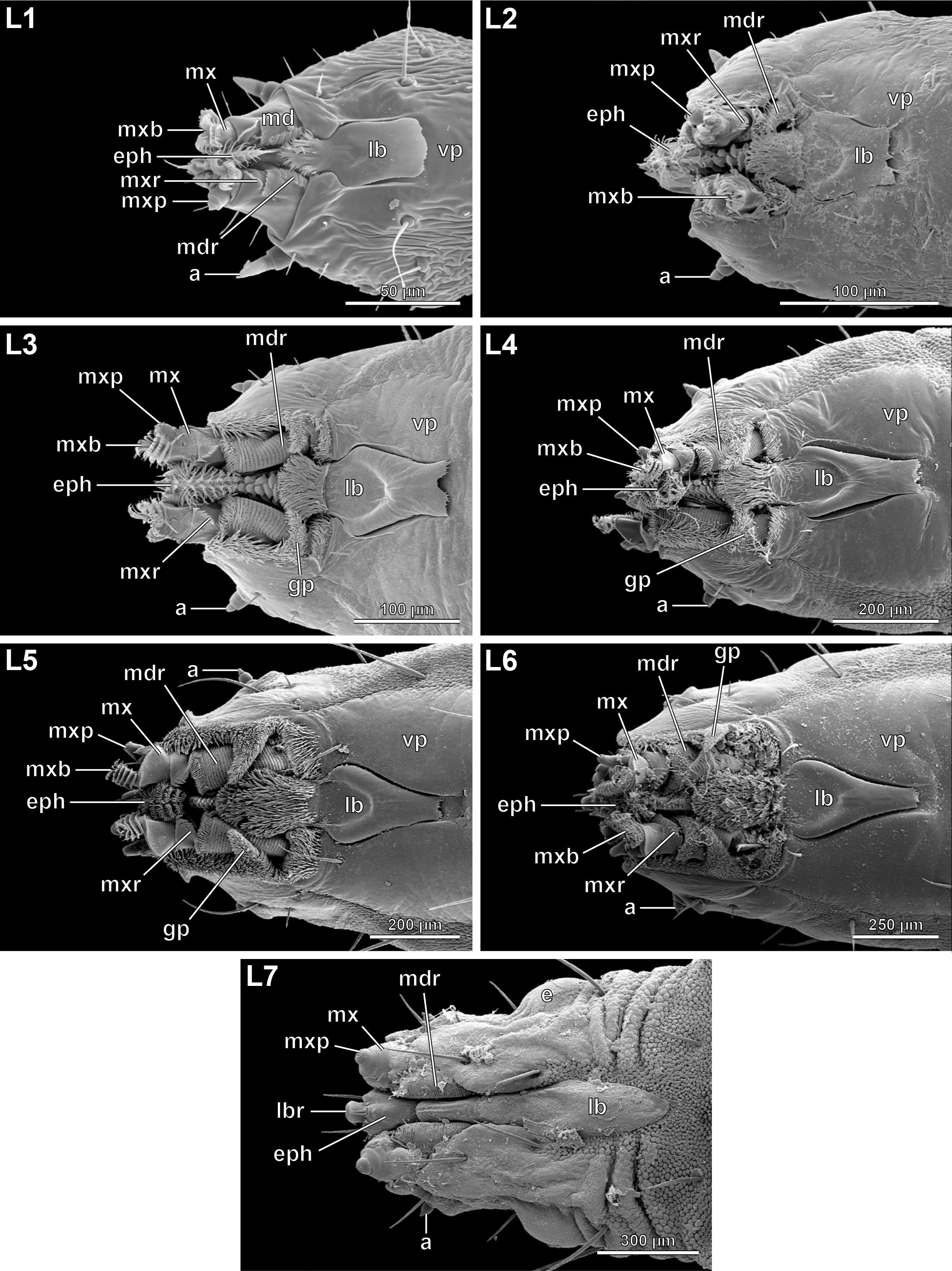

Supplement: Supplementary file 2 — Supporting Figure 2 ‐ Larval Head Ventral. [file JMOR-286-e70048-s001.jpg]

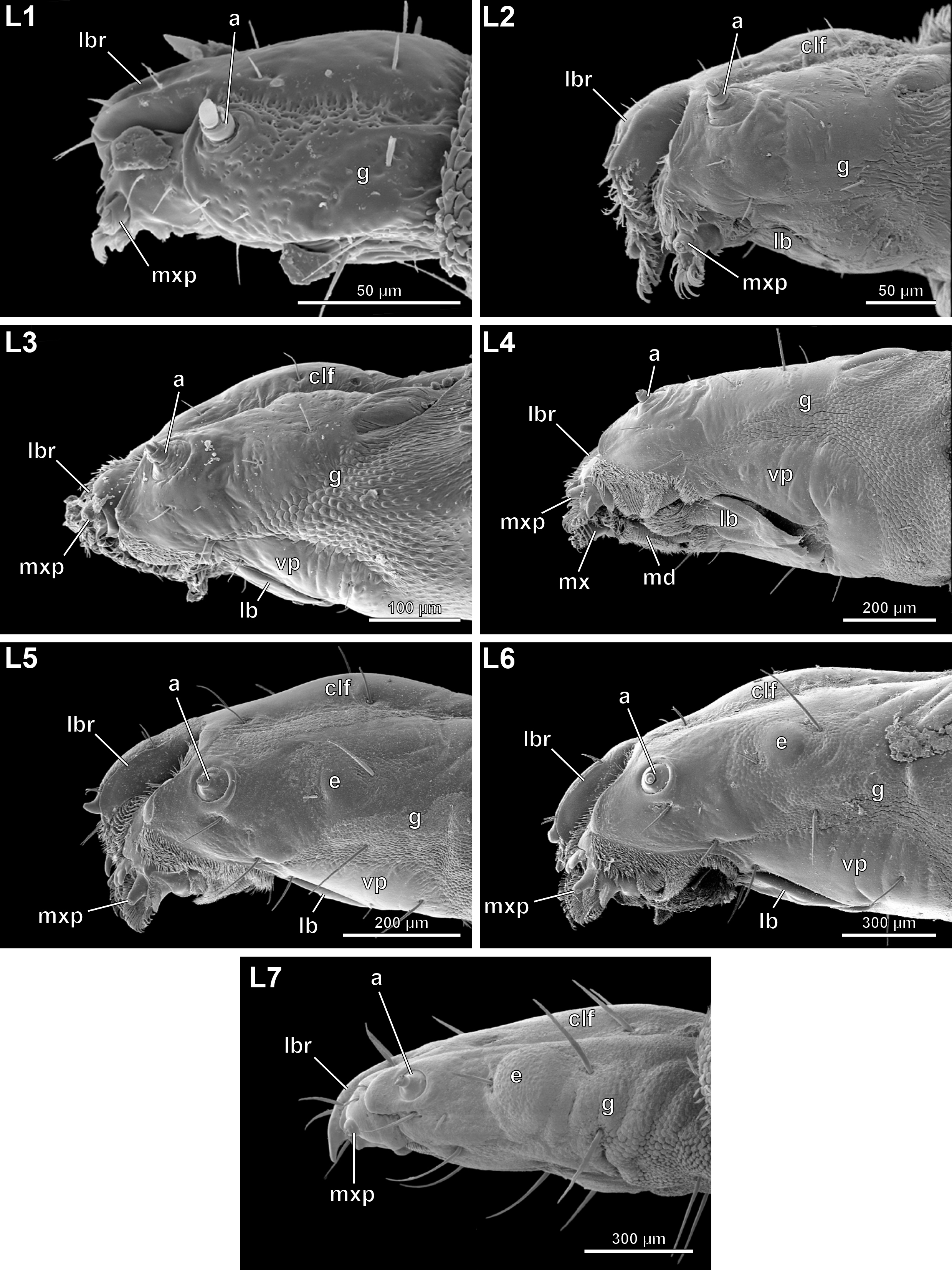

Supplement: Supplementary file 3 — Supporting Figure 3 ‐ Larval Head Lateral. [file JMOR-286-e70048-s006.jpg]

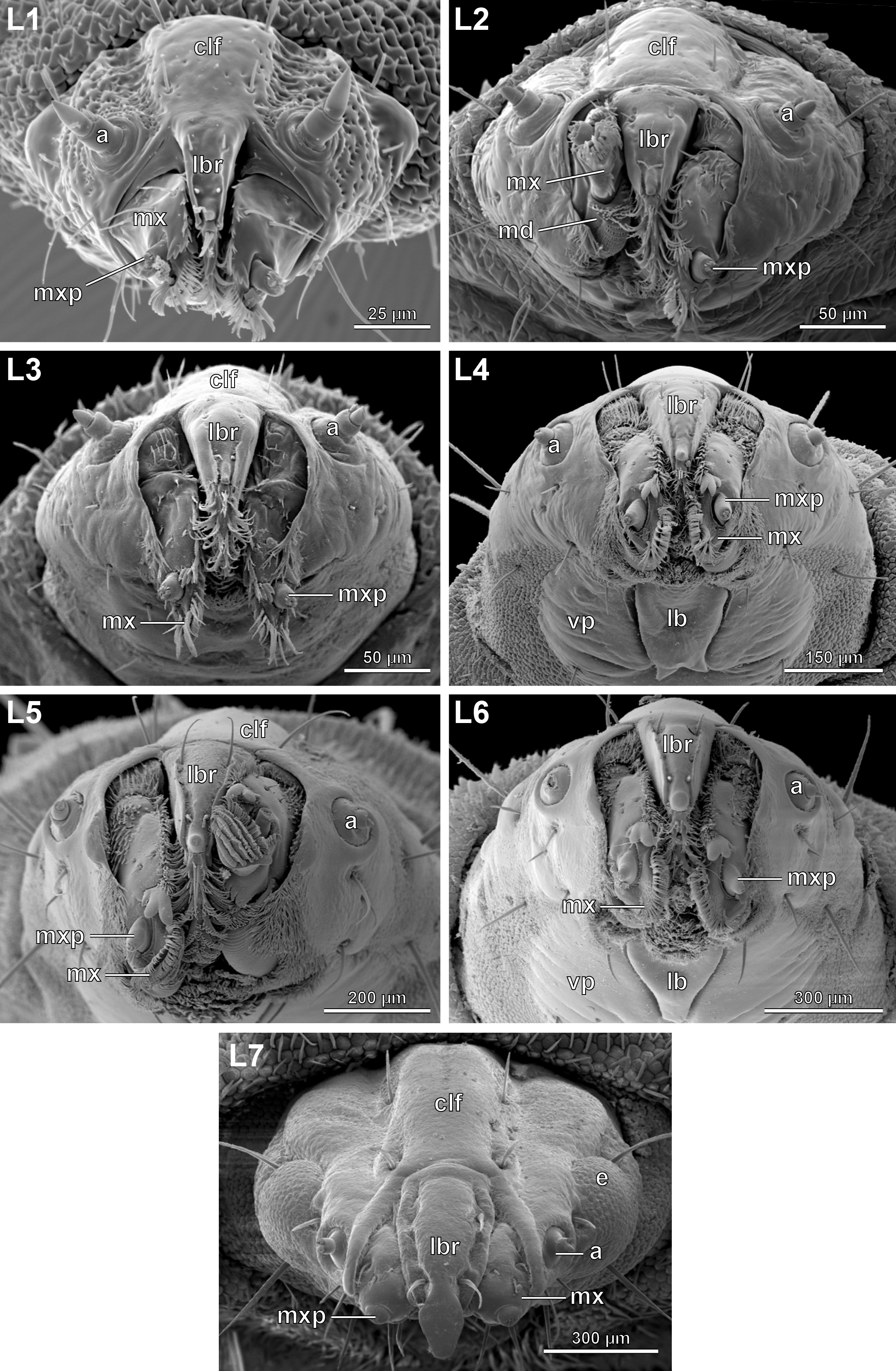

Supplement: Supplementary file 4 — Supporting Figure 4 ‐ Larval Head Frontal. [file JMOR-286-e70048-s005.jpg]
