# Supplementary material for: Transformations of Head Structures During the Larval Development of the Black Soldier Fly Hermetia illucens (Stratiomyidae, Diptera)
Source: J Morphol. 2025 Apr 11;286(4):e70048. doi: 10.1002/jmor.70048 (PMC11992506; doi:10.1002/jmor.70048)
Supplement: Supplementary file 6 — supmat. [file JMOR-286-e70048-s003.docx]

**7 Supplementary information**

**Supplementary Table 1**: Measurements (in mm) and classification of larval stages in previous studies. The values of Gligorescu et al., (2019) are rough approximations taken from their first figure.

| Larval instar | Measurements of previous publications | | | | | | |
| --- | --- | --- | --- | --- | --- | --- | --- |
|  | Head capsule length | Body length | Head capsule width | Head capsule width | Body length | Body length | Head capsule width |
|  | May 1961 | Schremmer 1986 | Kim et al., 2010 | Barros et al., 2019a, b | Barros et al., 2019b | Barros et al., 2019a | Gligorescu et al., 2019 |
| 1^st^ | 0.27 – 0.28 | 0.9 – 1.8 | 0.1 ± 0.02 | 0.05 – 0.08 | 1.00 – 2.63 | 1.6 ± 0.5 | [0.10] |
| 2^nd^ | 0.45 – 0.46 | 1.8 – 3.5 | 0.2 ± 0.04 | 0.09 – 0.24 | 1.64 – 4.00 | 2.7 ± 1.0 | [0.22] |
| 3^rd^ | 0.68 ± 0.09 | 3.5 – 7.5 | 0.4 ± 0.06 | 0.25 – 0.45 | 3.28 – 6.00 | 5.5 ± 2.0 | [0.44] |
| 4^th^ | 1.26 ± 0.09 | 8.0 – 11.0 | 0.6 ± 0.08 | 0.46 – 0.57 | 6.05 – 14.00 | 10.5 ± 2.2 | [0.67] |
| 5^th^ | 2.02 ± 0.36 | 11.0 – 14.0 | 0.9 ± 0.06 | 0.58 – 1.04 | 9.53 – 20.29 | 14.7 ± 2.6 | [0.80] |
| 6^th^ | 2.37 ± 0.29 | 14.0 – 17.0 | 1.1 ± 0.05 | 1.05 – 1.14 | 9.53 – 20.29 | 18.6 ± 1.7 | [1.08] |
| 7^th^ |  | 15.0 – 19.0 |  |  |  |  | [1.04] |
| Pupar-ium |  | 14.0 – 17.0 | 0.8 ± 0.06 | 0.73 – 0.86 | 15.00 – 22.29 |  |  |

**
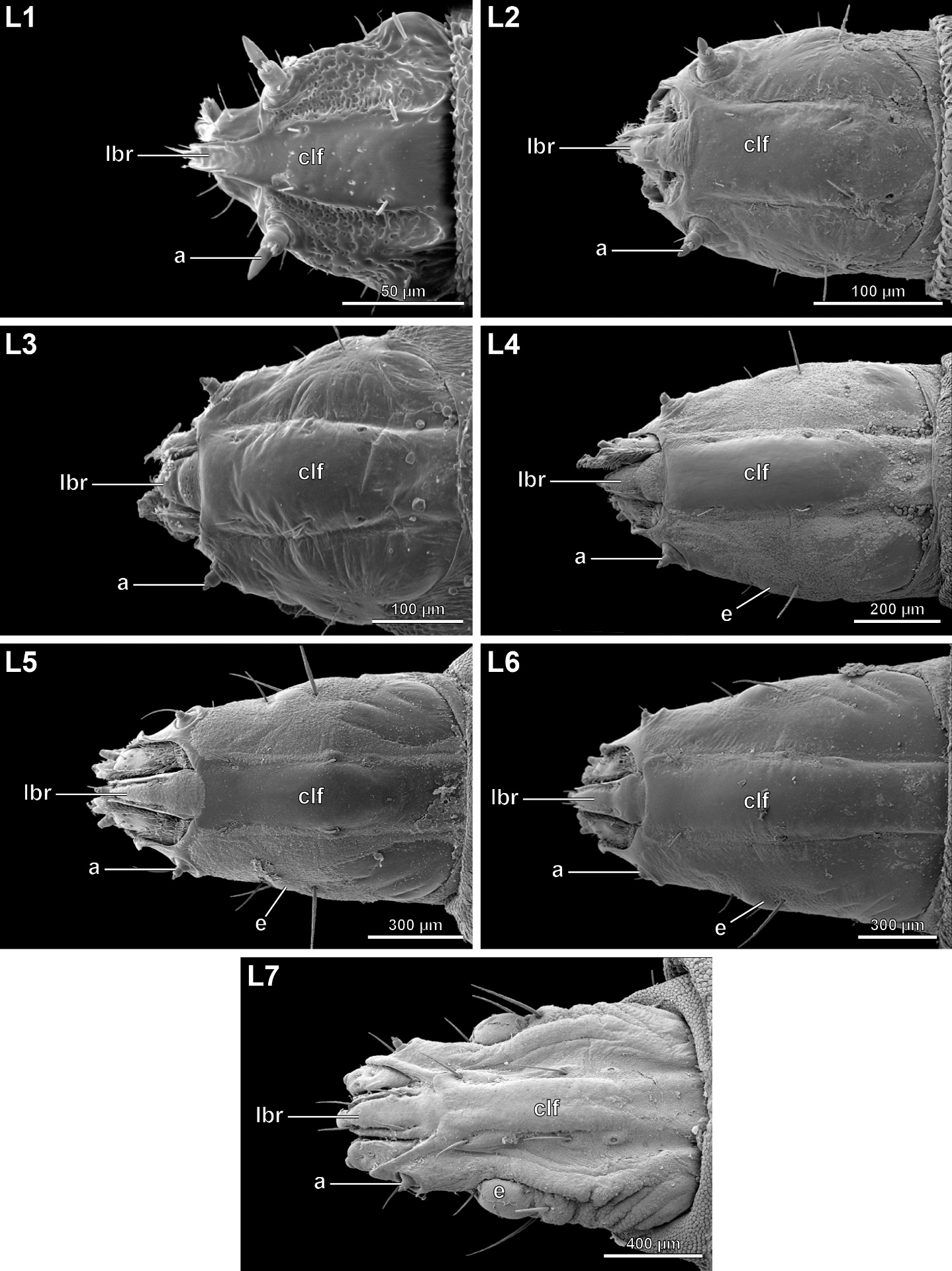
**

**Supplementary Figure 1:** *Hermetia illucens*, SEM images of all instars, dorsal view of the larval head. **L1**, 1^st^ instar. **L2**, 2^nd^ instar. **L3**, 3^rd^ instar. **L4**, 4^th^ instar. **L5**, 5^th^ instar. **L6**, 6^th^ instar. **L7**, 7^th^ instar. Abbreviations: a – antenna, clf – clypeofrontal region, e – eye, lbr – labrum.


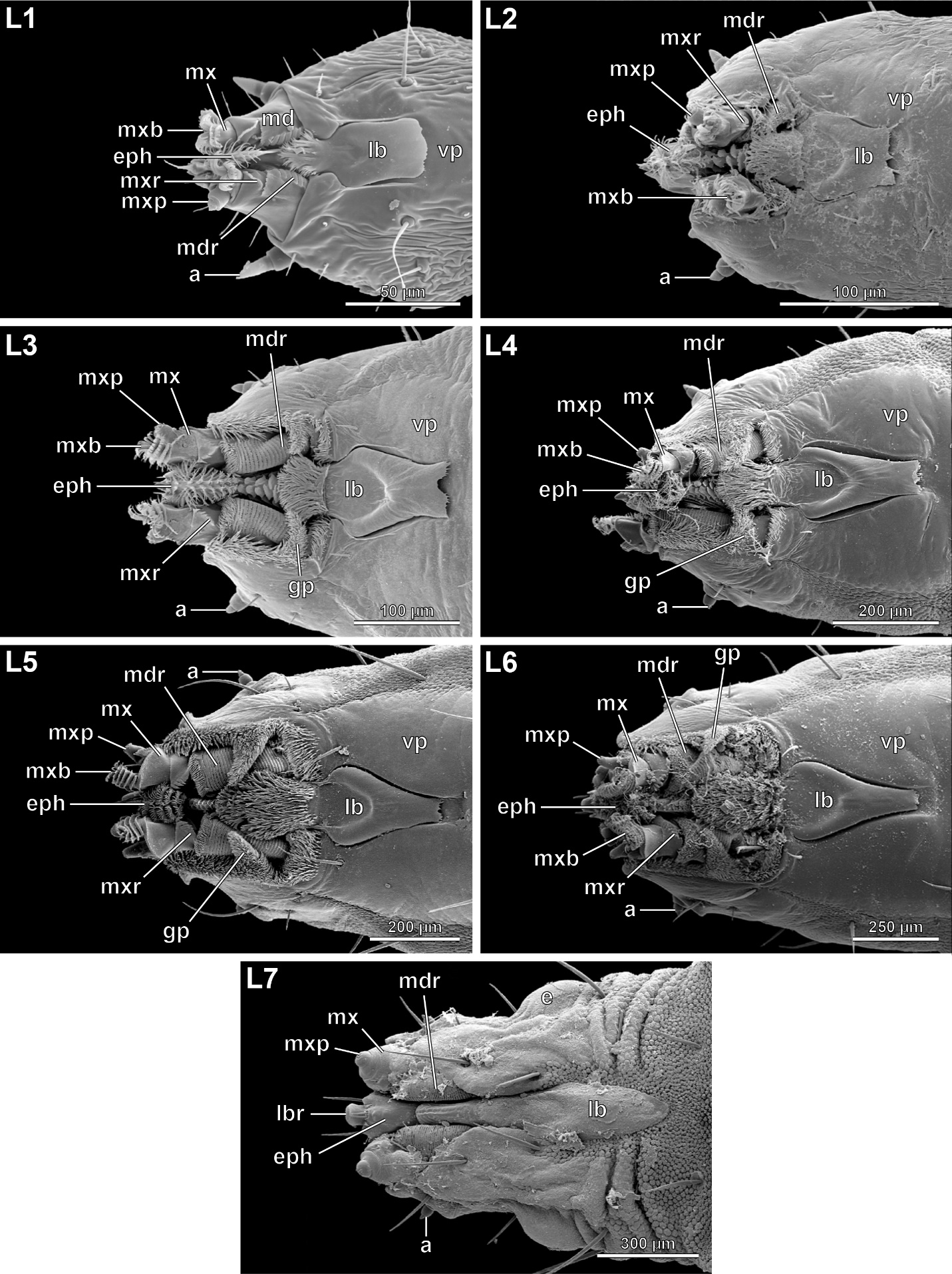


**Supplementary Figure 2:** *Hermetia illucens*, SEM images of all instars, ventral view of the larval head. **L1**, 1^st^ instar. **L2**, 2^nd^ instar. **L3**, 3^rd^ instar. **L4**, 4^th^ instar. **L5**, 5^th^ instar. **L6**, 6^th^ instar. **L7**, 7^th^ instar. Abbreviations: a – antenna, e – eye, eph – epipharynx, gp – triangular genal projection, lb – labium, lbr – labrum, md – mandibular part of the mandibulo-maxillary-complex, mdr – mandibular ridges, mx – maxillary part of the mandibulo-maxillary-complex, mxb – maxillary brush, mxp – maxillary palp, mxr – maxillary ridge, vp – ventral plate.


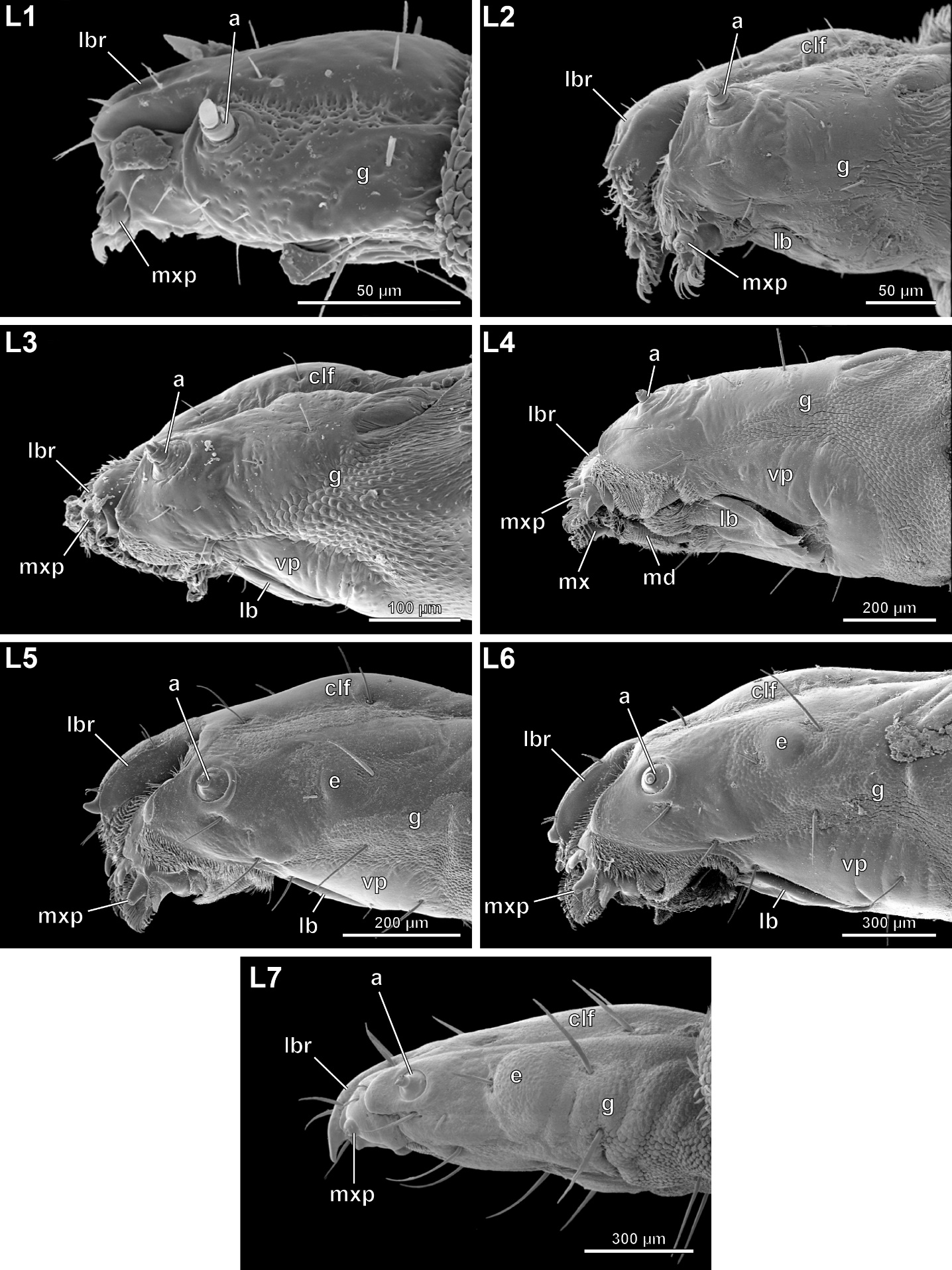


**Supplementary Figure 3:** *Hermetia illucens*, SEM images of all instars, lateral view of the larval head. **L1**, 1^st^ instar. **L2**, 2^nd^ instar. **L3**, 3^rd^ instar. **L4**, 4^th^ instar. **L5**, 5^th^ instar. **L6**, 6^th^ instar. **L7**, 7^th^ instar. Abbreviations: a – antenna, clf – clypeofrontal region, e – eye, g – gena, lb – labium, lbr – labrum, md – mandibular part of the mandibulo-maxillary-complex, mx – maxillary part of the mandibulo-maxillary-complex, mxp – maxillary palp, vp – ventral plate.

**
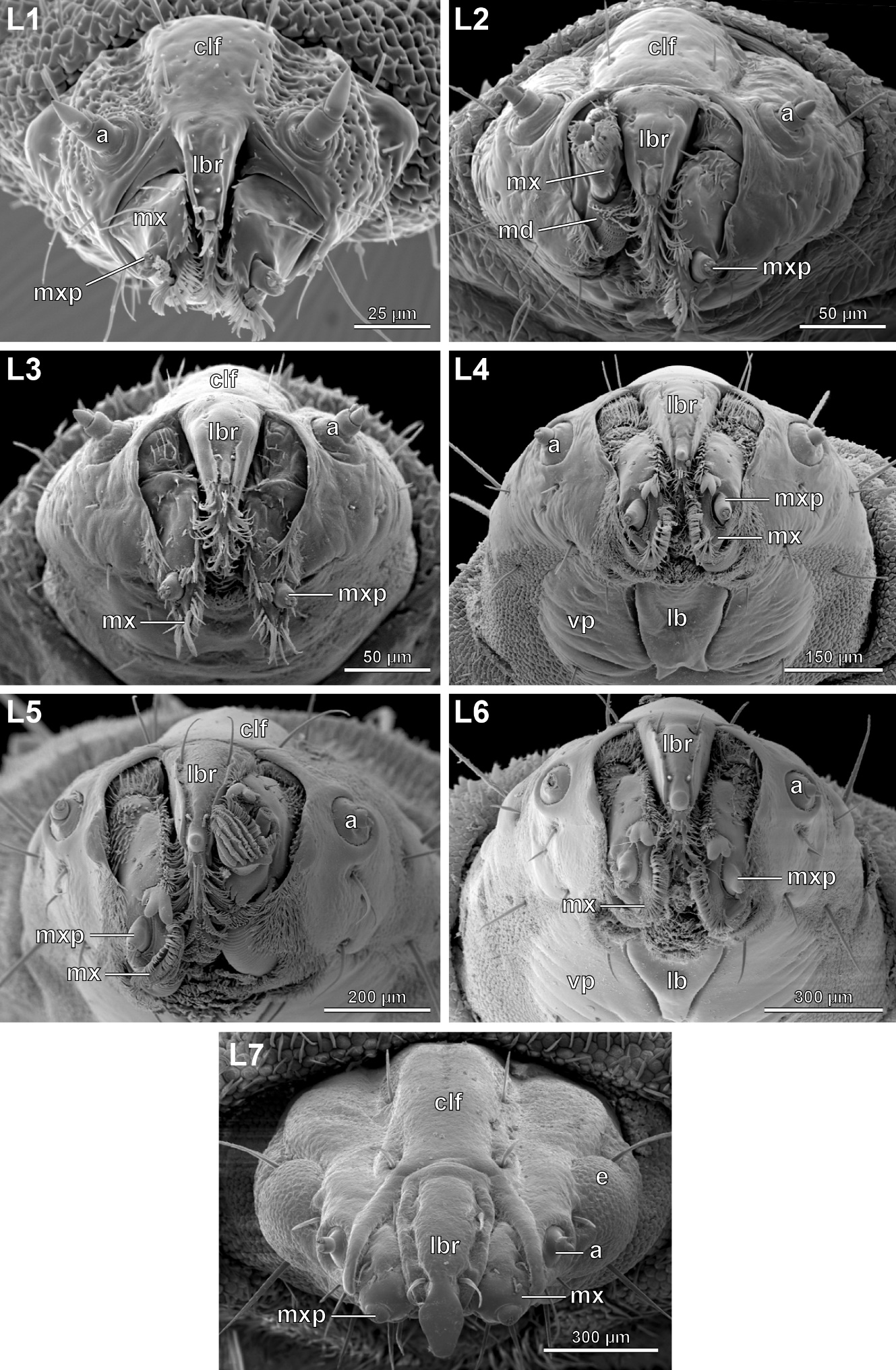
**

**Supplementary Figure 4:** *Hermetia illucens*, SEM images of all instars, frontal view of the larval head. **L1**, 1^st^ instar. **L2**, 2^nd^ instar. **L3**, 3^rd^ instar. **L4**, 4^th^ instar. **L5**, 5^th^ instar. **L6**, 6^th^ instar. **L7**, 7^th^ instar. Abbreviations: a – antenna, clf – clypeofrontal region, e – eye, lb – labium, lbr – labrum, md – mandibular part of the mandibulo-maxillary-complex, mx – maxillary part of the mandibulo-maxillary-complex, mxp – maxillary palp, vp – ventral plate.
